# Dynamic covalent nano-networks comprising antibiotics and polyphenols orchestrate bacterial drug resistance reversal and inflammation alleviation

**DOI:** 10.1016/j.bioactmat.2023.04.014

**Published:** 2023-04-18

**Authors:** Yuanfeng Li, Yin-Zi Piao, Hua Chen, Keqing Shi, Juqin Dai, Siran Wang, Tieli Zhou, Anh-Tuan Le, Yaran Wang, Fan Wu, Rujiang Ma, Linqi Shi, Yong Liu

**Affiliations:** a Translational Medicine Laboratory, The First Affiliated Hospital of Wenzhou Medical University, Wenzhou, Zhejiang, 325035, China; b Wenzhou Institute, University of Chinese Academy of Sciences, Oujiang Laboratory, Zhejiang Lab for Regenerative Medicine, Vision and Brain Health, Wenzhou, Zhejiang, 325001, China; c Key Laboratory of Functional Polymer Materials of Ministry of Education, Institute of Polymer Chemistry, College of Chemistry, Nankai University, Tianjin, 300071, China; d Department of Clinical Laboratory, The First Affiliated Hospital of Wenzhou Medical University, Wenzhou, Zhejiang, 325035, China; e Nano Institute, Phenikaa University, Yen Nghia, Ha Dong, Ha Noi, Viet Nam

**Keywords:** Biofilms, Dynamic covalent bonds, On-demand drug release, Resistance reversal, Biosafety

## Abstract

New antimicrobial strategies are urgently needed to meet the challenges posed by the emergence of drug-resistant bacteria and bacterial biofilms. This work reports the facile synthesis of antimicrobial dynamic covalent nano-networks (aDCNs) composing antibiotics bearing multiple primary amines, polyphenols, and a cross-linker acylphenylboronic acid. Mechanistically, the iminoboronate bond drives the formation of aDCNs, facilitates their stability, and renders them highly responsive to stimuli, such as low pH and high H_2_O_2_ levels. Besides, the representative **A1B1C1** networks, composed of polymyxin B1(**A1**), 2-formylphenylboronic acid (**B1**), and quercetin (**C1**), inhibit biofilm formation of drug-resistant *Escherichia coli*, eliminate the mature biofilms, alleviate macrophage inflammation, and minimize the side effects of free polymyxins. Excellent bacterial eradication and inflammation amelioration efficiency of **A1B1C1** networks are also observed in a peritoneal infection model. The facile synthesis, excellent antimicrobial performance, and biocompatibility of these aDCNs potentiate them as a much-needed alternative in current antimicrobial pipelines.

## Highlights

• Antimicrobial dynamic covalent nano-networks (aDCNs) were prepared via a one-pot three-component reaction.
• This methodology can be easily applied for the construction of other functional nano-networks for various applications.
• Iminoboronate bond drives the formation of aDCNs the release pharmacophores inside biofilms.
• Polyphenols down-regulates the expression of drug resistance genes in *Escherichia coli* and alleviates macrophage inflammation*.*

## Graphical abstract

**Figure:**
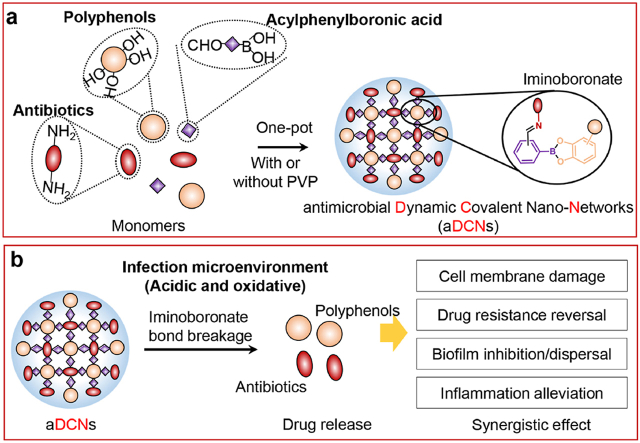

## 1 Introduction

Drug-resistant bacteria have posed an ever-increasing threat to global human health. An estimated 10 million people will die from infections caused by drug-resistant bacteria if no practical efforts to curtail bacterial resistance or new antibiotics are developed by 2050 [1,2]. Multiple mechanisms of drug resistance include target alterations, antibiotics-inactivating enzymes, membrane efflux pumps, and the formation of bacterial biofilms [[3], [4], [5], [6]]. Drug combinations [7], *via* either physical mixing or chemical bonding, of more than one antibiotic or antibiotic with an adjuvant [8] may offer a productive strategy to achieve multiple targets or inhibit the activity of enzymes that inactivate or pump out antibiotics. The flourish of preclinical drug combinations studies has failed mainly in clinical translation due to the different pharmacokinetic properties of the drugs in combination [9]. To maximize the clinical benefit of the drug combination, various drug hybrids composing two or more pharmacophores with distinct mechanisms of action have been developed [[10], [11], [12]]. Notably, most hybrids are conjugated via chemical bonds such as ester and amide, where the drug release is strongly limited by the bacterial hydrolases [7,13]. Although dynamic chemistry may provide more feasibility for drug targeting and release *via* an on-demand manner, there is still a distinct lack of facile ways to fabricate dynamic-covalent-bond-conjugated antibiotics hybrids.

Notably, dynamic covalent strategy offers enormous possibilities for generating materials using different building blocks [[14], [15], [16]]. Usually, these building blocks bond to each other via reversible dynamic covalent bonds, virtually encompassing various possible combinations, and allowing the construction of thermodynamically stable and adaptive processes owing to the dynamic interconversion of the constituents [[17], [18], [19]]. It has been witnessed that dynamic covalent strategy was applied in fields, such as drug discovery [17,20] and delivery [21], for applications ranging from cancer therapy to bacterial eradication [[22], [23], [24]]. In principle, an increase in the valency of the building blocks can lead to more complex topologies and functionalities.

**Scheme 1:**
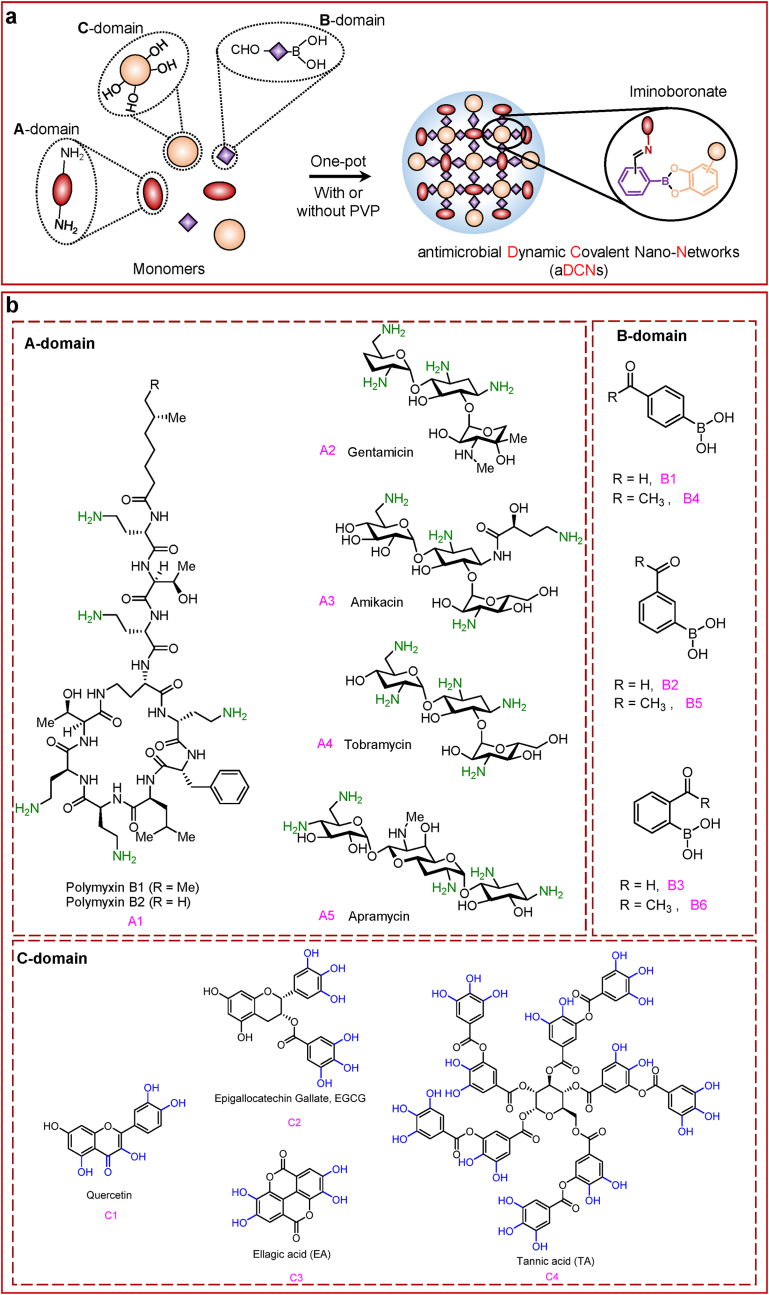
Design and formation of aDCNs. (**a**) Schematic illustration of the preparation of the iminoboronate-stabilized aDCNs. (**b**) Chemical structures of the **A**, **B**, and **C** domains used in fabricating aDCNs.

Herein, we report the facile fabrication of **a**ntimicrobial **D**ynamic **C**ovalent nano-**N**etworks (aDCNs) using a multicomponent reaction between multi-amine antibiotics (**A** domain, e.g., polymyxins and aminoglycosides), formyl-phenyl boronic acid (**B** domain), and polyphenols (**C** domain, e.g., catechols and pyrogallols), as illustrated in Scheme 1. Multi-amine antibiotics are commonly used in the clinic. For example, polymyxins efficiently eradicate Gram-negative bacteria by disrupting the membrane integrity. However, serious side effects, such as nephrotoxicity and neurotoxicity, may also occur due to the polyamine nature of these antibiotics. Worse still, bacteria are easy to mutate and being recalcitrant to conventional antibiotics, even the last resort of therapy, polymyxins [25]. Moreover, the positively charged antibiotics interact with the negatively charged biofilm matrix, limiting the penetration of antibiotics inside biofilms and, ultimately, the killing efficacy [5]. In our strategy, iminoboronate bonds will be formed orthogonally and act as dynamic cross-linkers between antibiotics and adjuvants, forming a highly branched nano-network structure. The amine groups of antibiotics are temporally protected via the formation of iminoboronate bonds. Iminoboronate bonds have been applied as the driving force to construct a myriad of functional structures [[26], [27], [28], [29], [30]]. Meanwhile, iminoboronate bonds are responsive to a wide range of intracellular parameters such as low pH, high glucose/glutathione/H_2_O_2_ levels, etc. [[31], [32], [33], [34], [35]] These aDCNs are expected to respond to a bacterial infection microenvironment with low pH and high H_2_O_2_ levels [[36], [37], [38]], releasing antibiotics and polyphenols that can synergistically address bacterial drug resistance and alleviate inflammation. Besides, the nanoscale nature endows these aDCNs with the capability to inhibit the formation of biofilms or eradicate the established biofilms better [5,[39], [40], [41]]. The temporal modification of amines on antibiotics may overcome their intrinsic cytotoxicity to normal tissues. These aDCNs could provide an efficient alternative pathway to overcome the drug resistance of bacteria and hold great potential in clinical translation.

## 2 Materials and methods

### 2.1 General procedure to prepare aDCNs

Before preparing the aDCNs, various stock solutions were freshly prepared, as illustrated in Table S1. To prepare **A1B1C1** nano-networks, PVP (6 mg) was dissolved in a carbonate buffer (pH 8.5, 50 mM, 2.21 mL), in which the stock solutions of 4-FPBA in DMSO (162 μL, **B1**) and polymyxin B in ultrapure water (300 μL, **A1**) were added at room temperature under a magnetic stirring (2500 rpm). Then, quercetin in DMSO (327 μL) was added dropwise at a rate of 1 drop/10 s with a final NH_2_/C=O/catechol molar ratio of 1:1:1 and a final concentration of polymyxin B of 1 mg/mL. The resulting suspension became milky upon adding polyphenols and kept being stirred at room temperature for 1 h. All other libraries were prepared essentially the same as the above-described procedure. The freshly prepared nanoparticles were kept in a fridge at 4 °C before further usage and tests.

### 2.2 Stability of A1B1C1 nano-networks

To study the stability of aDCNs, freshly prepared aDCNs in phosphate buffer (10 mM, pH 7.4), PBS with or without 10% fetal bovine serum, were kept at room temperature for 4 weeks. The size and zeta potential of aDCNs were measured on a Zetasizer Nano ZEN3600 (Malvern, UK).

### 2.3 Minimum inhibitory concentration (MIC) and bacterial concentration (MBC) determination

The MIC and MBC of various formulations were determined via our published procedure [42]. Briefly, 100 μL of antibiotics solutions, aDCNs (with an equivalent amount of antibiotics between 0 and 80 μg/mL) was applied to 100 μL of an *E. coli* WL5301 (or *S. aureus* Xen36) suspension in Lysogeny broth (LB) (or Tryptic Soy Broth, TSB) (2 × 10^5^ bacteria/mL). The MIC values were taken as the lowest antibiotics concentration at which bacterial growth was absent. Subsequently, the MBC values were determined by plating aliquots of suspensions with concentrations yielding no visible growth of bacteria on LB agar plates after being incubated for 24 h at 37 °C. The lowest concentration at which colony formation remained absent was taken as the MBC.

### 2.4 Biofilm growth inhibition

To inhibit the growth of biofilms, various formulations (**A1**, **A1C1**, and **A1B1C1** in PBS at an equal **A1** concentration of 64 μg/mL, 1 mL) were added to a confocal dish. The dish was kept at room temperature for 15 min statically. Then, a bacterial suspension of *E. coli* WL5301 (2 × 10^8^ bacteria/mL, 1 mL) was added to each dish and incubated at 37 °C for another 1 h. The resulting suspension was replaced with a 2 mL LB medium containing various formulations with an equal **A1** concentration of 64 μg/mL, incubated at 37 °C for 12 h. To visualize the biofilm growth, the supernatants were removed. Biofilms were stained with SYTO™ 9 for 15 min, fixed with a 4% fixative solution (Solarbio, Beijing, China), and observed on a Nikon A1 (Nikon, Japan) confocal laser scanning microscope using a 488 nm laser as excitation light and a filter of 500–550 nm. COMSTAT 2.1 ImageJ plugin was used to reconstruct the 3D images of biofilms, analyze the fluorescence intensity in each confocal plane, and construct the 3D project of the representative confocal plane at a depth of 6 μm from the biofilm bottom. Biofilms treated with PBS served as the control. Biofilm analysis: Biomass (μm^3^/μm^2^) is the volume of biomass divided by the area of view; Roughness Coefficient measures the variability in the height of the biofilm; Thickness (μm) is the height corresponding to the layer.

In a parallel experiment, biofilms were grown in 96-well plates according to our previous protocols [43]. Briefly, various formulations (**A1**, **A1C1**, and **A1B1C1** in PBS at an equal **A1** concentration of 64 μg/mL, 100 μL) were added to each well of 96-well plates. The plates were kept at room temperature for 15 min statically. Then, a bacterial suspension of *E. coli* WL5301 (100 μL, 2 × 10^8^ bacteria/mL) was added to each well and incubated at 37 °C for another 1 h. The resulting suspension was replaced with a 200 μL LB medium containing various formulations with an equal **A1** concentration of 64 μg/mL, incubated at 37 °C for 12 h. After that, supernatants were removed, and biofilms were washed with PBS (200 μL × 3). The remaining biofilms were dispersed with PBS (200 μL), homogenized by a pipet, serially diluted, and spread on LB agar plates. The bacterial colony grown overnight on the agar plates were recorded and compared.

To visualize the morphologies of the biofilms after various treatments, biofilms were gently rinsed with sterile water to remove unattached cells, fixed in 4% tissue fixative fluid for 15 min, dehydrated in a series of ethanol solutions (30-100%), and air-dried. The attached bacteria and formed biofilms on the surfaces were observed using a SU8010 microscope (Hitachi, Japan) with an accelerating voltage of 5.0 kV.

### 2.5 Assessment of eDNA release

For the analysis of eDNA, various formulations (**A1**, **A1C1**, **C1**, **A1B1C1** in PBS at an equal **A1** concentration of 32 μg/mL) were treated with the *E. coli* WL5301 followed the steps in biofilm growth inhibition. After treatment for 12 h, biofilms were dispersed and centrifuged twice at 14,000 rpm for 15 min and filtered by a 0.45 μm filter to remove any remaining bacteria. To quantify the eDNA concentration, each sample was incubated with propidium iodide (PI) (finalized at 1 μg/mL) for 15 min at 37 °C. The fluorescence emission was conducted at excitation 584 nm, and the emission at 612 was recorded.

In a parallel study, the eDNA solution (10 μL) was subjected to agarose gel electrophoresis. The gels were imaged on a VersaDoc 3000 imager (Bio-Rad, USA) and fluorescence intensity was quantified using ImageJ software (version 1.8.0).

### 2.6 Characterization of drug-resistance genes in E. coli

*E. coli* WL5301 were cultured with LB supplemented with various formulations (**A1**, **C1**, **A1C1**, **A1B1C1** in PBS at an equal **A1** concentration of 16 μg/mL, 1/2 MIC) for 12 h. The culture medium was removed, and total RNA was extracted from bacteria by Trizol reagent (Invitrogen). The reverse transcription was performed using PrimeScript^TM^RT reagent Kit (Takara Bio Inc) to get cDNA. SYBR Green Real-Time PCR Master Mix (Thermo Fisher Scientific) was used to perform the quantitative real-time polymerase chain reaction (qRT-PCR). 16s rDNA was used as the internal control, and the 2^−ΔCt^ method was used to calculate the relative expression of genes (the PBS-treated group was normalized to 1). The sequences of the primers used in this study are listed in Supplementary Table S2.

### 2.7 Mature biofilm dispersal

To grow mature biofilms, briefly, a bacterial suspension of *E. coli* WL5301 (1 mL, 2 × 10^8^ bacteria/mL) was added to each dish and incubated at 37 °C for another 1 h to allow the adhesion of bacteria. The bacterial suspension was removed carefully, and the dishes were washed with PBS (1 mL × 3). Then LB medium (2 mL) was added to each dish and incubated at 37 °C for 12 h. To study interactions of aDCNs with bacterial biofilms, a suspension of PBS containing **A1**, **A1C1**, and **A1B1C1** at an equal **A1** concentration of 64 μg/mL was added to the resultant biofilms, incubated at 37 °C for 4 h. The supernatants were removed, and the biofilms were stained with SYTO™ 9 and PI for 15 min, fixed with a 4% fixative solution (Solarbio, Beijing), and observed on a Nikon A-1 (Nikon, Japan) confocal laser scanning microscope. COMSTAT 2.1 ImageJ plugin was used to reconstruct the 3D images of biofilms, analyze the fluorescence intensity in each confocal plane, and construct the 3D project of the representative confocal plane at a depth of 6 μm from the biofilm bottom. Biofilms treated with PBS served as the control. Biofilm analysis: Biomass (μm^3^/μm^2^) is the volume of biomass divided by the area of view; Roughness Coefficient measures the variability in the height of the biofilm; Thickness (μm) is the height corresponding to the layer.

### 2.8 Scavenging free radicals in vitro

The hydroxyl radical (•OH) and superoxide radical (^•^O_2_‾) scavenging activity of nano-networks were studied. Briefly, •OH was produced by a Fenton reaction between CuSO_4_ and H_2_O_2_ (10 μL of 2 mM CuSO_4_ and 1 mL of 0.1 mM H_2_O_2_), and different samples (10 μL, 0.5 mg/mL) were added and incubated at 37 °C for 10 min •OH was measured by adding 3,3′,5,5′-tetramethylbenzidine (TMB, 10 μL of 1 mM) to the above mixture, and their UV–Vis spectra were recorded on a UV-1900i spectrometer.

^•^O_2_‾ was generated by mixing xanthine oxidase (50 μL, 0.05 U/mL) and xanthine (900 μL, 0.6 mM) in PBS at 37 °C for 30 min. Different samples (10 μL, 0.5 mg/mL) were added to the above solution and incubated for another 30 min, followed by the addition of hydroethidine (10 μL, 50 μg/mL). The amount of superoxide anion concentration was determined by measuring the fluorescent intensity of ethidium (the oxidation product of hydroethidine by superoxide radical) using a Hitachi F-4600 fluorescence spectrophotometer (Tokyo, Japan) at 470 nm excitation and emission at 610 nm.

### 2.9 Scavenging intracellular reactive oxygen species

RAW 264.7 cells were seeded in 12-well plates (10^5^ cells/well) and were cultured overnight at 37 °C for 12 h. The cells were then treated with 1 mM *tert*-butyl hydroperoxide (TBHP) for 4 h. After being washed with PBS three times, the cells were treated with nano-networks or other formulations for another 24 h. After being stained with H_2_DCFDA and DAPI, the cells were washed repeatedly with PBS and were imaged using a fluorescence microscope. The fluorescence was measured at 527 nm with a multi-well plate reader via an excitation at 492 nm.

### 2.10 In vitro anti-inflammation

BMDM cells were used to study the anti-inflammation of different formulations. The BMDM cells (2 × 10^5^ cells/well) were seeded in 12-well plates and incubated overnight. Cells were stimulated by LPS (5 μg/mL) for 1 h and treated with different formulations. The cells were cultured for another 24 h, and the supernatant was used for ELISA analysis according to the user's guide.

### 2.11 Hemolysis rate evaluation

Fresh mice blood (1 mL) was suspended in PBS (10 mL), centrifuged at 4000 rpm for 15 min, and washed with PBS (10 mL × 5) to separate red blood cells (RBCs). Then, the RBCs were resuspended in PBS (10 mL) at a volume concentration of 4%. Various formulations in PBS (100 μL) at different concentrations (64–2048 μg/mL) were added to RBC suspension in PBS (4%, 100 μL). To evaluate the hemolysis of aDCNs, the resultant RBC suspension (400 μL) was mixed with PBS containing **A1**, **A1C1**, or **A1B1C1** with **A1** concentration ranging from 0 to 1000 μg/mL, incubated at 37 °C for 3 h and then centrifuged at 3000 rpm for 15 min. The absorbance of the supernatant was measured at 545 nm on a plate reader. RBCs treated with Triton-X and PBS were used as the positive and negative controls, respectively. The hemolysis was calculated using the following equation:

$$Hemolysis\;\left(\%\right)=\left({OD}_{test}\;–\;{OD}_{neg}\right)/\left({OD}_{pos}\;–\;{OD}_{neg}\right)\times 100\%$$

Where OD_*test*_, OD_*neg*_, and OD_*pos*_ are the OD values of samples, the negative control, and the positive control, respectively.

### 2.12 In vivo bacterial eradication and anti-inflammation

Institute of Cancer Research (ICR) mice (female, 18–20 g) were intraperitoneally injected with *E. coli* WL5301 in PBS (100 μL, 5 × 10^7^ bacteria) and randomly divided into five groups (12 mice per group). After 2 h, mice were intraperitoneally injected with PBS (100 μL, group A, control), **A1** in PBS (100 μL, group B), **C1** in PBS (100 μL, group C), **A1C1** in PBS (100 μL, group D), or **A1B1C1** in PBS (100 μL, group E) at an **A1** dose of 2.5 mg/kg. The blood was collected through the eye of mice on day 2 and day 5 post-treatments, cytokines in blood were quantified by ELISA kits according to the user guide, and the main blood parameters were measured. After that, PBS (3 mL) was intraperitoneally injected into each mouse, abdomens were massaged for 3 min, and lavage fluids were collected. Part of the peritoneal fluid was serially diluted and spread on LB agar plates for CFU counting. The other part of the peritoneal fluid was used to analyze the cytokines according to the user guide. The major organs (heart, liver, spleen, lung, kidney) were fixed in 10% formalin, decalcified, embedded in paraffin, and subjected to hematoxylin and eosin (H&E) staining.

### 2.13 Catheter implant infection model

The implant infection model was constructed following the previous publications [[44], [45], [46]]. Briefly, ICR mice (female, 20–25 g) were anesthetized, shaved, and disinfected. The back of the mice was dissected to expose subcutaneous layers in a sterile environment. Subsequently, sterile thermoplastic polyurethane catheters (5 mm × 5 mm) were inserted, and the wound was sutured. *E. coli* WL5301 in PBS (10^6^ bacteria mL^−1^) was injected onto the implants subcutaneously. Two days later, the infected mice were randomly allocated into four groups (12 mice per group): PBS (100 μL, control), **A1** in PBS (100 μL), **C1** in PBS (100 μL), **A1C1** in PBS (100 μL), or **A1B1C1** in PBS (100 μL) at an **A1** dose of 2.5 mg/kg were injected into the infection area of each mouse. The infection area of each mouse was monitored post-surgery. Three days after different treatments, six mice per group were euthanized using pentobarbital for CFU enumeration and cytokine analysis. All other mice were euthanized at day 7 post-treatments, the blood was collected through the eye of mice after 24 h, cytokines in blood were quantified by ELISA kits according to the user guide, and the main blood parameters were measured. The implants and would tissues were collected for CFU enumeration and cytokine determination. The major organs (heart, liver, spleen, lung, kidney) were fixed in 10% formalin, decalcified, embedded in paraffin, and subjected to H&E staining.

### 2.14 Statistical analysis

All data are shown as mean ± s.d. from at least triplicate conditions unless otherwise indicated. Each experiment was repeated at least three times independently unless otherwise indicated. Statistical analyses included two-sided unpaired Student's t-test and one-way ANOVA. The *P* values of less than 0.05 were considered to indicate the statistical significance and are labeled in the results. Statistical analyses were done with GraphPad Prism 8 and Microsoft Excel 2019 software.

## 3 Results and discussion

### 3.1 Iminoboronate bonds drive the formation of aDCNs

Our efforts to prepare the aDCNs started by mixing polymyxin B1 (**A1**) and 2-formylphenylboronic acid (**B1**) in a carbonate buffer (pH 8.5). Surprisingly, when the quercetin (**C1**) was added dropwise, a brown flocculent precipitate was formed immediately, even under vigorous magnetic stirring. We then used polyvinylpyrrolidone (PVP) as an adjuvant to stabilize the as-formed networks (Table S1). PVP is a water-soluble and biocompatible polymer widely used in nanoparticle preparation [47]. The introduction of PVP could facilitate the stabilization of the nanoparticle surface and regulate the reaction rate via hydrogen bonding with polyphenols. We found that the fine nano-networks were formed within minutes when we mixed the four components (Movie S1). Instead, a blurry suspension was observed when mixing **A1** with **C1** in the absence of the **B** domain (Movie S2). Within 1 h, the suspension became transparent, demonstrating that no nano-networks were formed without the **B** domain. The as-prepared **A1B1C1** nano-networks had a hydrodynamic size of around 136 nm with a narrow distribution (polydensity index, PDI ∼ 0.147) and a negatively charged surface (Fig. S1). **A1**, **B1**, and **C1** were selected due to the following reasons. First, polymyxins (**A1**) are considered the last resort of therapy to treat multi-drug-resistant bacteria. While the highly dense positive charge of polymyxins often leads to nephrotoxicity and neurotoxicity. Therefore, this study aims to find a facile way to prevent the emergence of drug resistance and its side effects while maintaining its bactericidal efficacy. Second, **B1** and **C1** are the simplest structure in their corresponding libraries. Third, in our previous study, quercetin (**C1**) was found efficient in preventing the development of polymyxin drug resistance in Gram-negative bacteria [48].

**Fig. 1:**
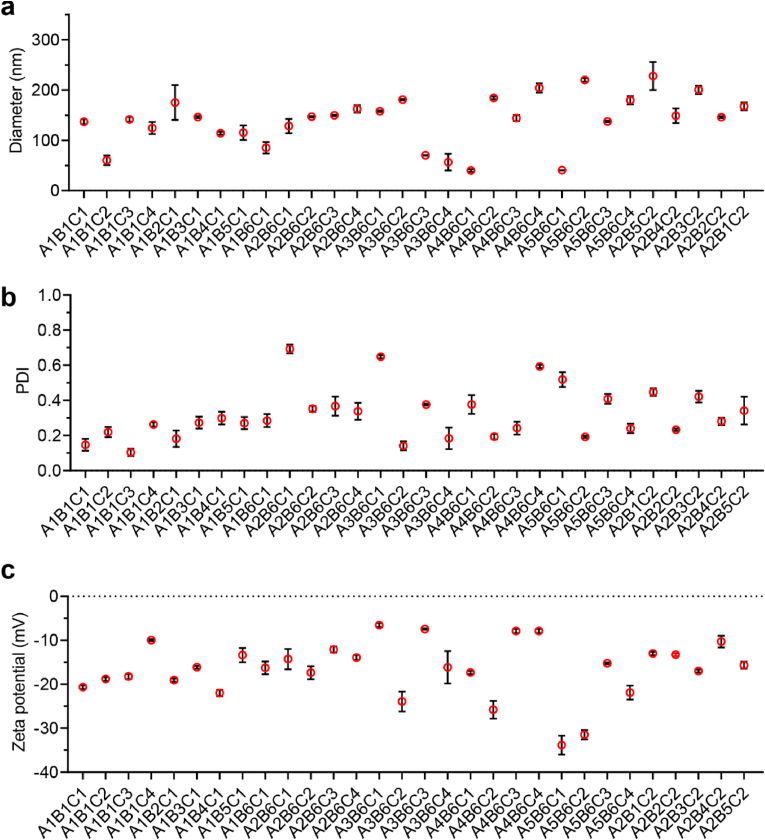
Screening the formation of aDCNs. (**a**) Hydrodynamic diameter, (**b**) polydensity index (PDI), and (**c**) zeta potential of the aDCNs with various components.

This success in the fabrication of nano-networks encouraged us to investigate the combinations of various domains, as illustrated in Scheme 1. Random combinations of various domains offered nano-networks with diameters ranging from 50 to 200 nm, PDIs less than 0.5, and negatively charged surfaces (Fig. 1). In our study, thirty DCNs in total were prepared. These results collectively demonstrate the potential of this methodology in fabricating antibiotics-containing DCNs in a facile way. To further demonstrate the power of this methodology, DCNs composing other amine-containing (bio)molecules and polyphenols are being developed in our lab for various applications.

**Fig. 2:**
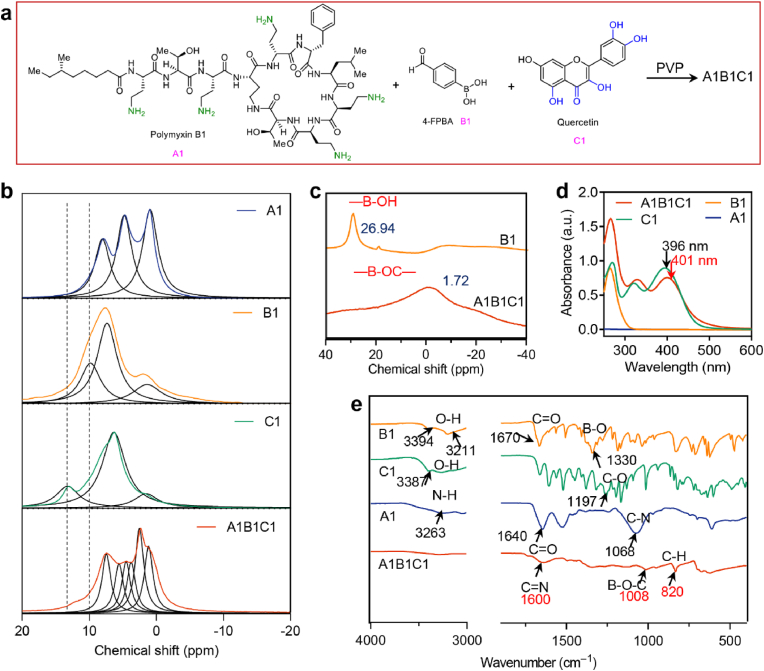
Iminoboronate bond drives the formation of aDCNs. (**a**) Chemical structures of the monomers used to fabricate **A1B1C1** nanoparticles. (**b**) ^1^H solid-state NMR spectra of **A1**, **B1**, **C1**, and **A1B1C1**, suggesting the formation of iminoboronate bonds. (**c**) ^11^B NMR spectra of **B1** and **A1B1C1** nano-networks in D_2_O. (**d**) UV–vis absorption of the different monomers and **A1B1C1** nano-networks in water. (**e**) FT-IR spectra of various formulations.

We then used **A1**, **B1**, and **C1** as the models to elucidate the chemical bond formation during nano-networks fabrication (Fig. 2a). As determined by solid-state nuclear magnetic resonance (NMR) spectra (Fig. 2b), the peaks of **B1** and **C1** at chemical shifts above 10 ppm disappeared when the **A1B1C1** nano-networks formed. Similar results were observed in the solution-state NMR spectra (Fig. S2). Also, the chemical shift of **B1** in ^11^B NMR spectra at 26.94 ppm was replaced with 1.72 ppm in **A1B1C1** nanonetworks (Fig. 2c), suggesting the transformation from phenylboronic acid to boronate. Besides, the absorption peak of **C1** red-shifted to 401 nm in **A1B1C1** nanonetworks (Fig. 2d). Furthermore, the stretching vibration of C=O occurred at 1640 cm^−1^ and the stretching vibration of B–O occurred at 1330 cm^−1^ of **B1** disappeared after the reaction in their corresponding Fourier-transform infrared (FT-IR) spectra. Meanwhile, the vibration of C=N occurred at 1600 cm^−1^ and vibration of B–O–C occurred at 1008 cm^−1^ were detected in the as-formed nano-networks (Fig. 2e). These results collectively suggest that imino-boronate bonds are formed during the fabrication of nano-networks.

### 3.2 Nano-networks characterizations

**Fig. 3:**
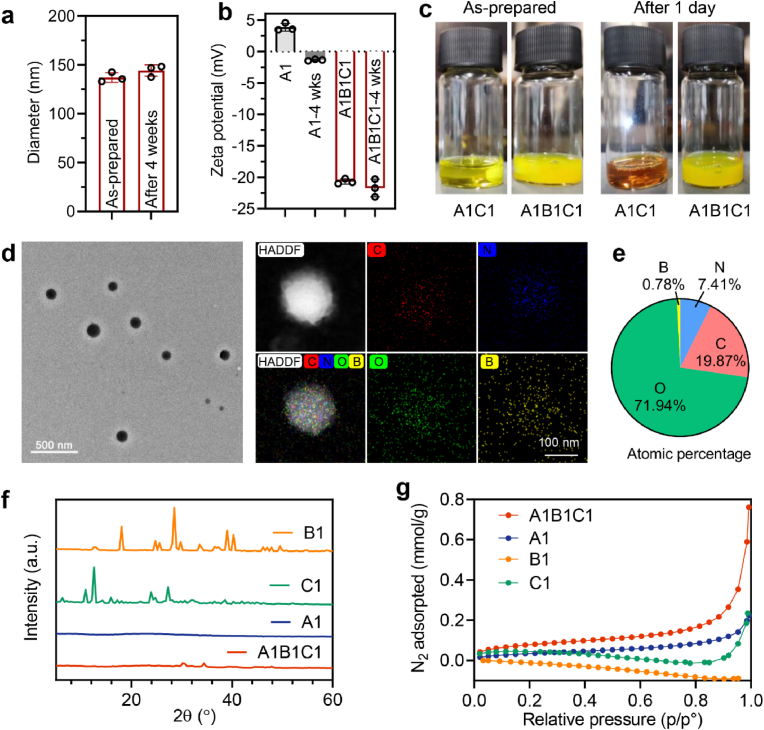
Characterizations of **A1B1C1** nano-networks. (**a**) Diameters of the as-prepared **A1B1C1** nano-networks and nano-networks stored at room temperature for 4 weeks. (**b**) Zeta potential of the as-prepared **A1** in solution and **A1B1C1** nano-networks and **A1** in solution and nano-networks stored at room temperature for 4 weeks. (**c**) Photographs of the as-prepared **A1C1** and **A1B1C1** nano-networks and the **A1C1** and **A1B1C1** nano-networks stored at room temperature for 1 day. (**d**) Representative TEM image and EDS elemental mapping of the **A1B1C1** nano-networks. (**e**) Percentage of elements in the **A1B1C1** nano-networks. (**f**) X-ray powder diffraction (XRD) of **A1B1C1** nano-networks. (**g**) N_2_ adsorption isotherm of **A1B1C1** nano-networks.

Then we characterized the formed nano-networks. The as-formed nano-networks were remarkably stable, and no notable variations in size and zeta potential of nano-networks were observed over the 4-week observation period in phosphate buffer (PB), phosphate-buffered saline (PBS) with or without fetal bovine serum (FBS) (Fig. 3a, 3b, and S3). Whereas **A1** alone in solution was not stable due to its peptide nature, as demonstrated by the fact that **A1** becomes negatively charged in solution (Fig. 3b). In the absence of **B1**, **A1C1** turned brownish yellow within 24 h incubation in an ambient oxygen atmosphere at room temperature (Fig. 3c), probably due to the oxidation of **C1**. By contrast, a minor color change was noticed for the **A1B1C1** nano-networks under the same conditions (Fig. 3c). UV–Vis absorption spectra (Fig. S4) also demonstrate the oxidation of **C1** in solution. On the contrary, **A1B1C1** nano-networks were negligibly affected by the ambient oxygen atmosphere and temperature, suggesting the formation of boronate bonds could stabilize the catechol structure in **C1** and prevent it from being oxidized (Fig. S5). Of note, nanoformulations also enhanced the thermal stability of **A1**, as demonstrated in the thermogravimetric test (Fig. S6). Our results demonstrated that the addition of **B1** and **C1** domains could neutralize the positive charge on **A1**, which may facilitate the stability of **A1** and alleviate its cytotoxicity caused by its highly dense positive charge. The diameters of nanonetworks changed significantly and rapidly when exposed to an acidic and/or H_2_O_2_ environment (Fig. S7), likely due to the break of imino-boronate bonds. Further characterizations showed that the **A1B1C1** nano-networks have a spherical morphology and elements such as carbon, nitrogen, oxygen, and boron homogenously distributed over the networks, as illustrated in the transmission electron microscopy (TEM) (Fig. 3d) and scanning electron microscopy (SEM) images (Fig. S8). X-ray diffraction analysis (XRD) indicated that the **A1B1C1** nano-networks possess an amorphous structure (Fig. 3f). And N_2_ adsorption isotherm curves showed the porous nature of **A1B1C1** nano-networks (Fig. 3g).

### 3.3 aDCNs are efficient in killing drug-resistant pathogens

With all the aDCNs in hand, we then studied the bactericidal efficacy of the aDCNs, as summarized in Table S3. Undoubtedly, PVP, **B** and **C** domains demonstrated negligible antimicrobial efficacy with MIC and MBC values higher than 500 μg/mL against the clinically isolated *Escherichia coli* (*E. coli*) and multi-drug resistant *Staphylococcus aureus* (*S. aureus* Xen36) [43,49]. The clinically isolated *E. coli* is a polymyxin-resistant strain with a MIC value of 32 μg/mL. The encapsulation of **A1** in nano-networks could maintain the bactericidal efficacy of the original **A1**. Similar results were found in aminoglycoside-based aDCNs, which can maintain or lower the MIC and MBC values of the original aminoglycosides against *S. aureus* Xen36. Polyphenols can enhance the bactericidal efficacy of conventional antibiotics as an adjuvant due to their performance in downregulating the expression of drug-resistance genes [50]. To elucidate the mechanisms, we studied the expression of representative drug-resistant genes, such as plasmid-mediated colistin resistance gene (*mcr-1*) [51], aminoglycoside resistance gene (*str-A*) [52], and tetracycline resistance gene (*tet-A*) [52], after the treatment of **A1B1C1** nano-networks via quantitative reverse transcriptase-polymerase chain reaction (qRT-PCR) (Fig. S9). These drug-resistance genes were notably downregulated after being treated with **A1B1C1** nano-networks. Notably, the downregulation of drug-resistance genes did not lead to notably lower MIC and MBC values against bacteria, likely due to the loaded drugs not ultimately released, which was also observed in our previous studies [42,53].

### 3.4 A1B1C1 nano-networks inhibit the formation of *E. coli* biofilms and eradicate the pre-formed biofilms

**Fig. 4:**
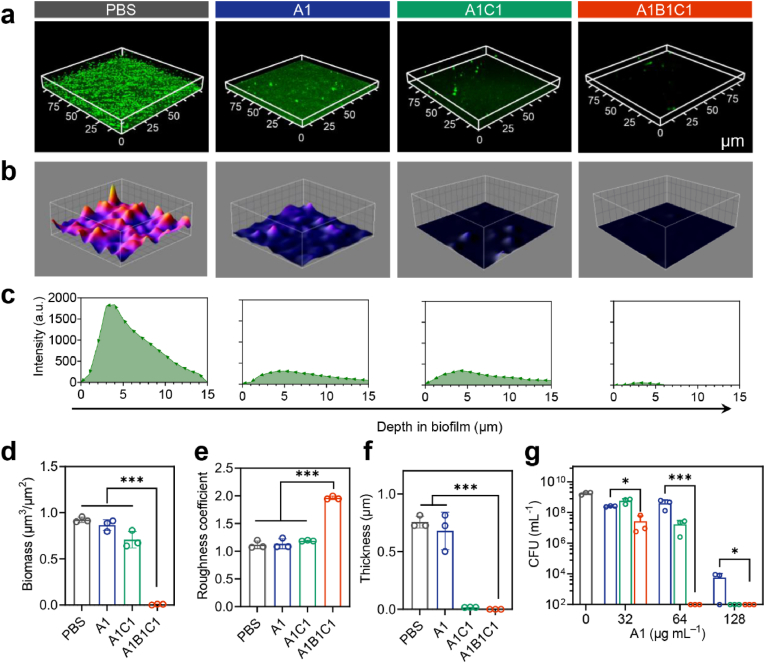
Inhibition of *E. coli* WL5301 biofilm formation using **A1B1C1** nano-networks. (**a**) Representative CLSM images of the biofilms after exposure to PBS, **A1** or **A1C1** in solution, and **A1B1C1** nano-networks. Bacteria embedded in biofilms are represented as green. (**b**) 3D projection of the CLSM plane at 6-μm from the bottom of biofilms after various treatments. (**c**) Quantified fluorescence intensity in each CLSM plane as a function of depth in biofilm. (**d**) Biomass, expressed as volume of biomass divided by the area of view, (**e**) Roughness coefficient, and (**f**) Average thickness of the stack quantified using COMSTAT 2.1 ImageJ plugin. (**g**) Quantified CFU of *E. coli* WL5301 biofilms after various treatments at different concentrations. Data were expressed as mean ± standard deviation (SD) over triplicates.

Next, the efficacy of aDCNs in inhibiting biofilm formation and eradicating mature biofilms was investigated. A biofilm is a cluster of bacteria and their self-produced extracellular polymeric matrixes [[54], [55], [56]]. Biofilm matrixes protect the embedded inhabitants from microenvironmental variations and antibiotic exposure [[57], [58], [59]]. In this regard, biofilms account for over 80% of bacterial infections and contribute significantly to the drug resistance of bacteria [5,41]. Although various nanotechnology-based agents have been developed to combat bacterial biofilms, it is still challenging to eliminate the biofilms formed by drug-resistant bacteria, especially Gram-negative strains [39]. Two strategies, biofilm inhibition and dispersion [60], are commonly used. Normally, biofilm inhibition was realized by modifying the surfaces of substrates with superb hydrophilic polymers such as polyethylene glycol (PEG) or polyzwitterions [61,62]. We first investigated the biofilm inhibition ability of our **A1B1C1** nano-networks using the clinically isolated *E. coli* WL5301 as a model strain. There were considerable *E. coli* biofilms formed on the phosphate-buffered saline (PBS)-treated surfaces. The treatments of **A1** and **A1C1** notably inhibited the growth of biofilms, as demonstrated by their corresponding confocal laser scanning microscopy (CLSM) images, reconstructed 3D projections, and quantified fluorescence intensity (Fig. 4a, 4b). The thickness and biomass of the biofilms treated with **A1** and **A1C1** in solution were significantly reduced (Fig. 4c). **A1B1C1** nano-networks achieved the best biofilm formation inhibition efficacy, as we observed from the quantified biomass, roughness coefficient, and thickness of the biofilms after various treatments (Fig. 4d and e). Alternatively, the biofilm formation inhibition efficacy of the various formulations was also investigated via the colony-forming unit (CFU) counting method, as demonstrated in Fig. 4g, where a concentration-dependent biofilm inhibition performance was observed for all the tested formulations. At an equal **A1** dose, **A1B1C1** nano-networks exhibited superior biofilm inhibition efficacy compared to **A1** or **A1C1** in solution. At an **A1** concentration of 64 μg/mL, **A1B1C1** nano-networks could completely inhibit biofilms' growth. Similar results were observed via the SEM images of *E. coli* after the treatment of **A1B1C1** nano-networks (Fig. S10). We then analyzed the gene expression of *fimH* [63], the major detriment of type 1 fimbriae for adherence. The treatment of **A1B1C1** networks could notably downregulate the expression of the *fimH* gene in the *E. coli* biofilms (Fig. S11). Moreover, **A1B1C1** nano-networks also prevent eDNA production in *E. coli* biofilms (Figs. S12 and S13). These results collectively suggested that our **A1B1C1** nano-networks can prevent biofilm formation by killing bacteria, inhibiting bacterial adhesion toward a substratum, and eDNA generation. Different from the conventional mechansim of using hydrophilic polymer-coated surface to prevent the attachment of bacteria for a long term, our strategy may provide a solution to kill bacteria and prevent the initial biofilm formation.

**Fig. 5:**
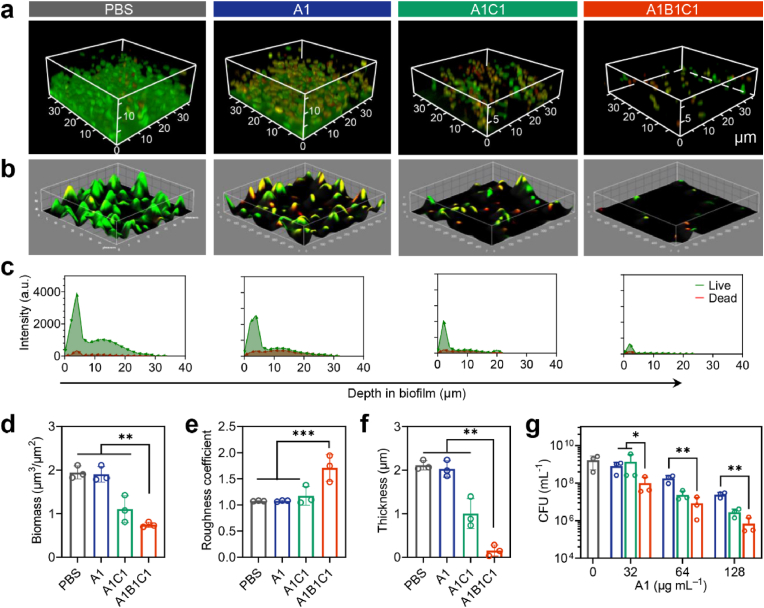
Dispersal of *E. coli* WL5301 biofilm using **A1B1C1** nano-networks. (**a**) Representative CLSM images of the *E. coli* WL5301 biofilms after exposure to PBS, **A1** or **A1C1** in solution, and **A1B1C1** nano-networks and stained with SYTO9 and PI. Live bacteria embedded in biofilms are represented as green, and dead bacteria are represented as red. (**b**) 3D projection of the CLSM plane at 6-μm from the bottom of biofilms after various treatments. (**c**) Quantified fluorescence intensity in each CLSM plane as a function of depth in biofilm. (**d**) Biomass, expressed as volume of biomass divided by the area of view, (**e**) Roughness coefficient, and (**f**) Average thickness of the stack quantified using COMSTAT 2.1 ImageJ plugin. (**g**) Quantified CFU of *E. coli* WL5301 biofilms after various treatments at different concentrations. Data were expressed as mean ± standard deviation (SD) over triplicates.

To eliminate the pre-formed *E. coli* biofilms, nano-networks were mixed with 1-day-old biofilms, and then the biofilms were stained with SYTO9/PI for CLSM observation. As illustrated in Fig. 5, the treatments of **A1** and **A1C1** in solution partially dissociated the mature biofilms, and numerous dead (red) bacteria were observed (Fig. 5a–c). Also, **A1C1** in solution demonstrated better efficacy in biofilm dispersal than **A1** in solution, possibly due to the adjuvant effect of **C1**. Meanwhile, **A1B1C1** nano-networks demonstrated the best biofilm elimination performance, as we observed from the quantified biomass, roughness coefficient, and thickness of the biofilms after various treatments (Fig. 5d and e).In a parallel study, we also quantified the CFU of biofilms after various treatments. **A1B1C1** nano-networks are far better than **A1** and **A1C1** in eliminating biofilm bacteria, especially at a relatively lower **A1** concentration. For instance, neither **A1** nor **A1C1** in solution efficiently killed bacteria embedded in biofilms at an **A1** concentration of 32 μg/mL. Meanwhile, **A1B1C1** nano-networks achieved 90% killing efficacy at the equal **A1** concentration. Of note, a 3-log reduction in CFU was achieved when treating the biofilms with **A1B1C1** nano-networks at an A1 concentration of 128 μg/mL (Fig. 5g), suggesting their potential in clinical translation. Similar results were also observed via the SEM images of *E. coli* after the treatment of **A1B1C1** nano-networks (Fig. S14).

### 3.5 A1B1C1 nano-networks alleviate ROS and cellular inflammation

**Fig. 6:**
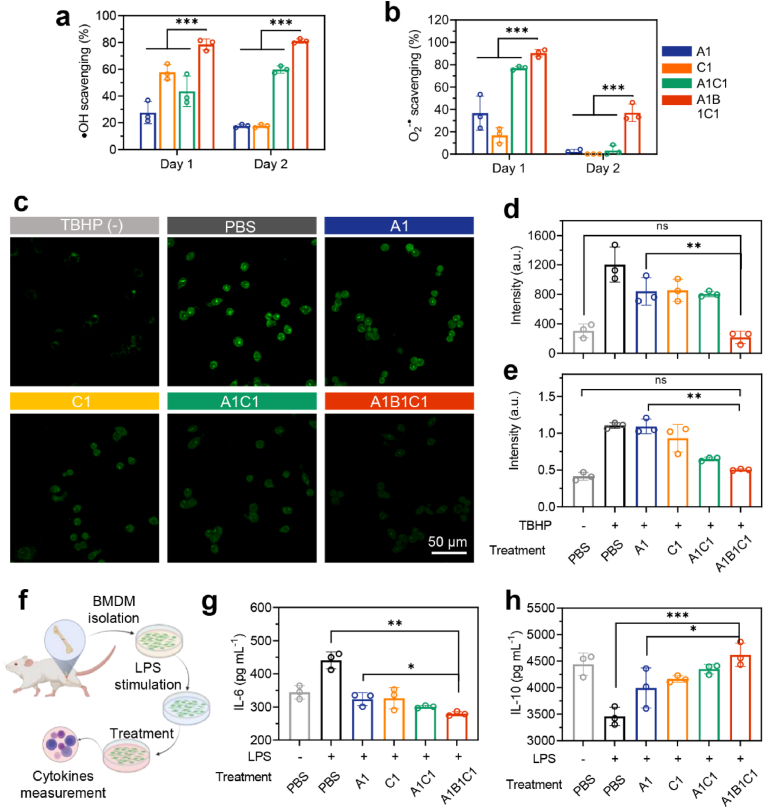
ROS scavenge of **A1B1C1** nano-networks. (**a**) •OH scavenging ability of various formulations measured at different storage time intervals. (**b**) •O_2_^−^ scavenging ability of various formulations measured at different storage time intervals. (**c**) CLSM images showing the intracellular ROS scavenging of various formulations in the TNHP-treated macrophages. (**d**) Quantified fluorescence intensity from the CLSM images as shown in **c**. (**e**) Quantified fluorescence of TBHP in RAW 264.7 macrophages after various treatments at an equal **A1** concentration of 64 μg/mL. (**f**) Workflow of the LPS-induced BMDM inflammation model. (**g**) IL-6 expression in the BMDMs after various treatments. (**f**) IL-10 expression in the BMDMs after various treatments. ns represents no significant difference. ns represents no significance, *p < 0.05, **p < 0.01, ***p < 0.001, and ****p < 0.0001, one-way ANOVA.

Polyphenols serve as useful anti-inflammatory adjuvants because they scavenge ROS, thus terminating the subsequent chain reactions on/in cells. To determine the ROS scavenging efficiency of our **A1B1C1** nano-networks, we first tested the clearance of •OH and •O_2_^−^ in the presence of **A1B1C1** nano-networks using UV–Vis and fluorescence spectrometry. As shown in Fig. 6a, 6b and Fig. S15, **C1** and **A1B1C1** nano-networks demonstrated similar and highest •OH and •O_2_^−^ scavenging efficiency, suggesting that the incorporation of **C1** in nanoformulation will not affect its ROS scavenging ability. The advantage of our **A1B1C1** nano-networks was that the ROS scavenging ability of **C1** was maintained mainly even after being kept at room temperature for 24 h, likely since **A1B1C1** nano-networks could stabilize **C1** and prevent the oxidation of **C1** in solution.

Similar results were found in a *tert*-butyl hydroperoxide (TBHP)-induced inflammatory macrophage model, where intracellular ROS was notably eliminated when the inflamed cells were treated with **A1B1C1** nano-networks, as observed via their corresponding CLSM images (Fig. 6c). Of note, **A1B1C1** nano-networks exhibited far better ROS-elimination ability than **C1** or **A1C1** in solution, likely due to the poor solubility of **C1** or **A1C1** in water and subsequent poor internalization efficiency. Consistently, quantified fluorescence intensity from either CLSM images (Fig. 6d) or the microplate reader (Fig. 6e) showed the prior ROS-elimination ability of **A1B1C1** nano-networks compared to all other treatments. In detail, interleukin 6 (IL-6) [64] and interleukin 10 (IL-10) [65] were selected as the representative cytokines to indicate the inflammation states of the macrophages after various treatments. Compared to other treatments, both IL-6 and IL-10 in the lipopolysaccharide (LPS)-treated bone-marrow-derived macrophages (BMDMs) were notably neutralized after the **A1B1C1** nano-networks treatment (Fig. 6f–h). These results indicated that the treatment of **A1B1C1** nano-networks could efficiently regulate the inflammation of the LPS-treated macrophages, which might benefit bacterial eradication via immune intervention [66].

### 3.6 A1B1C1 nano-networks eliminate bacteria and inflammation in an abdominal murine infection model

**Fig. 7:**
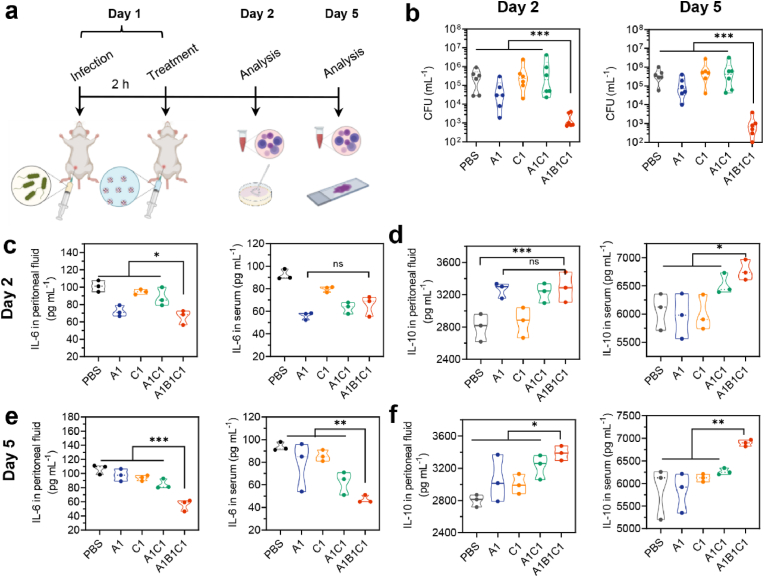
**A1B1C1** nano-networks eradicate bacteria and alleviate inflammation in a peritoneal infection model. (**a**) Workflow of the *in vivo* experiment. (**b**) Quantified CFU of *E. coli* WL5301 in the peritoneal infections after various treatments at an equal **A1** concentration of 64 μg/mL on day 2 and day 5 post-treatment. (**c**) IL-6 expression in the peritoneal fluid and serum on day 2 after various treatments at an equal **A1** concentration of 64 μg/mL (**d**) IL-10 expression in the peritoneal fluid and serum at day 2 after various treatments at an equal **A1** concentration of 64 μg/mL (**e**) IL-6 expression in the peritoneal fluid and serum on day 5 after various treatments at an equal **A1** concentration of 64 μg/mL (**f**) IL-10 expression in the peritoneal fluid and serum on day 5 after various treatments at an equal **A1** concentration of 64 μg/mL ns represents no significance, *p < 0.05, **p < 0.01, ***p < 0.001, and ****p < 0.0001, one-way ANOVA.

Then a murine abdominal infection model was constructed using *E. coli* WL5301, and the infected mice were intraperitoneally injected with various treatments (Fig. 7a). **A1B1C1** nano-networks demonstrated the best antimicrobial efficacy compared to all other treatments. A 2.5-log reduction in CFU was achieved when the mice were treated with **A1B1C1** nano-networks (Fig. 7b) compared to the PBS-treated control group. Moreover, IL-6 in the bacteria-infected mice was neutralized after the **A1B1C1** nano-networks treatment, while IL-10 was notably upregulated (Fig. 7c, 7d). This regulation of inflammation cytokines is likely due to the eradication of bacteria at the infection sites [24]. Indeed, the expression of IL-6 and IL-10 did not show significant differences between the treatment of **A1** and **A1B1C1** on day 2 post-treatment. In contrast, significant differences appear on day 5 post-treatment, when most bacteria have been eradicated. Moreover, H&E staining of major organs after treatment with various treatments indicated the lack of obvious pathological abnormalities (Fig. S16). Similarly, major blood parameters of mice treated with **A1B1C1** nano-networks showed minor variations compared to PBS (Fig. S17). It therefore indicated the desired biocompatibility of NCs with minimal systemic toxicity.

### 3.7 A1B1C1 nano-networks eliminate biofilm-associated implant infection

**Fig. 8:**
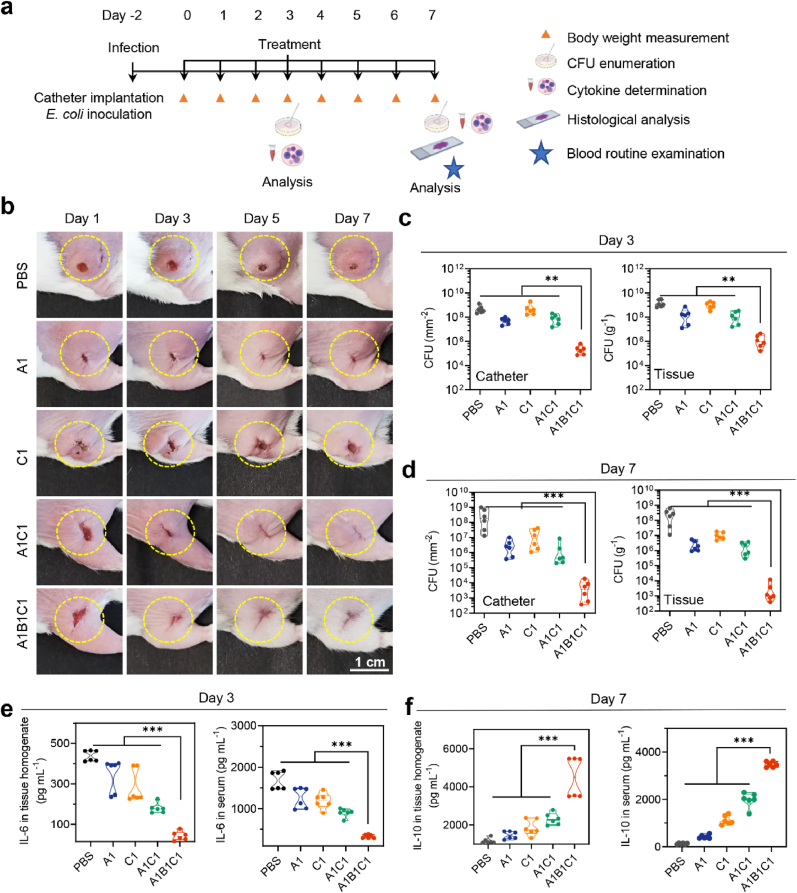
**A1B1C1** nano-networks eliminate biofilm-associated implant infection. (**a**) Workflow of the *in vivo* experiment. (**b**) Representative images showing the wounds at different time points over the 7-d observation period post-treatment at an equal **A1** concentration of 64 μg/mL (**c,d**) Quantified CFU from the catheters and wound tissues after various treatments on day 3 (**c**) and day 7 (**d**) post-treatment at an equal **A1** concentration of 64 μg/mL. IL-6 (**e**) and IL-10 (**f**) expression in the peritoneal fluid and serum on day 3 and day 7 after various treatments at an equal **A1** concentration of 64 μg/mL **p < 0.01, ***p < 0.001, one-way ANOVA.

We then constructed a subcutaneous catheter implant infection model and evaluated the bactericidal efficacy of our **A1B1C1** nano-network in eradicating bacteria and alleviating inflammation, as illustrated in Fig. 8a. The treatment of **A1B1C1** nano-networks showed the property to accelerate wound closure, superior compared to all other treatments (Fig. 8b). Besides, **A1B1C1** nano-networks demonstrated the best antimicrobial efficacy compared to all other treatments. A 3.5-log reduction in CFU in the catheters and wound tissues was achieved when the mice were treated with **A1B1C1** nano-networks (Fig. 8c) on day 3 post-treatment compared to the PBS-treated control group. On day 7, A 5-log reduction in CFU in the catheters and wound tissues was achieved with **A1B1C1** nano-networks (Fig. 8d) on day 7 post-treatment. The expression of IL-6 was largely neutralized after the **A1B1C1** nano-networks treatment, while IL-10 was notably upregulated (Fig. 8e and f). Similarly, major blood parameters of mice treated with **A1B1C1** nano-networks showed minor variations compared to PBS (Fig. S18). And the body weight of mice was negligibly affected by all the treatments (Fig. S19). Besides, H&E staining of major organs after treatment with various treatments indicated the lack of obvious pathological abnormalities (Fig. S20). These results collectively suggested that our **A1B1C1** nano-networks could efficiently eradicate the bacteria and alleviate inflammation in the implant infection model without notable side effects to the normal tissues.

### 3.8 A1B1C1 nano-networks ameliorate the cytotoxicity of A1

**Fig. 9:**
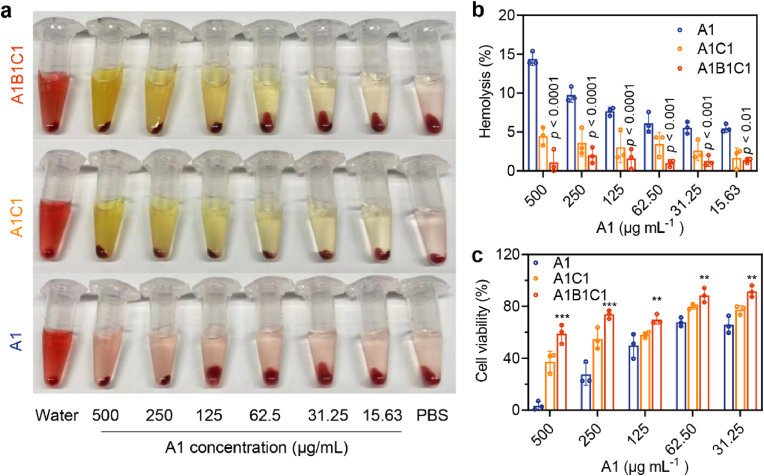
**A1B1C1** nano-networks ameliorate the cytotoxicity of **A1**. (**a**) Photographs of the red blood cells after various treatments at different concentrations. (**b**) Quantified hemolysis rate of red blood cells after various treatments at different concentrations. Red blood cells treated with PBS served as the negative control, and the cells treated with water served as the positive control (100% hemolysis rate). (**c**) Viability of HEK293 cells after various treatments at different concentrations. The cell viability of HEK293 treated with PBS was set as 100%. **p < 0.01, ***p < 0.001, one-way ANOVA.

Typically, the clinical application of polymyxins is primarily plagued by their cytotoxicity caused by their dense positive surface charge. Various strategies, such as encapsulating or entangling polymyxins into nanoparticles, have been developed to minimize cytotoxicity and target the infection sites [67]. In our design, the amino groups on the polymyxin or other antibiotics are temporally protected by the imine bonds. Therefore, the **A1B1C1** nano-networks have negatively charged surfaces that allow the stability of nano-networks in solution and minimize the cytotoxicity caused by positively charged materials [68]. To evaluate the cytotoxicity of our **A1B1C1** nano-networks, firstly, their hemolysis rate was evaluated using murine red blood cells. Negligible hemolysis was observed even though the red blood cells were treated with **A1B1C1** nano-networks at an **A1** concentration of 500 μg/mL. In contrast, **A1** in solution induced significant hemolysis of red blood cells (Fig. 9a and b). Similarly, in human embryonic kidney (HEK293) cells, free **A1** in solution showed remarkable cytotoxicity, whereas **A1B1C1** nano-networks could significantly alleviate the cytotoxicity of free **A1** (Fig. 9c). Therefore, our **A1B1C1** would efficiently alleviate the original cytotoxicity of free **A1** while maintaining or strengthening its bactericidal efficiency.

## 4 Conclusion

In summary, we have constructed the aDCNs composing the amine-containing antibiotics, acylphenylboronic acid, and polyphenols via a facile one-pot reaction in minutes. The iminoboronate bond in aDCLs drove the formation of aDCLs and facilitated the stabilization of aDCNs. Meanwhile, the nanoformulation of aDCLs notably alleviated the intrinsic cytotoxicity of the multiple amine-containing antibiotics and prevented the polyphenols from being oxidized. The representative **A1B1C1** nano-networks demonstrated superior antimicrobial efficacy compared to the free antibiotics in preventing biofilm formation and eliminating mature biofilms. Also, **A1B1C1** nano-networks orchestrated bacterial killing and inflammation alleviation in a murine peritoneal infection model. This facile methodology of preparing aDCLs and their efficiency in therapy may provide a much-needed alternative for treating drug-resistant bacteria-induced infections in this 'post-antibiotics' era.

## Ethics approval and consent to participate

The animal experimental protocols were reviewed and approved by the Institutional Animal Care and Use Committee, Wenzhou Institute, University of Chinese Academy of Sciences (No. WIUCAS21071223).

## CRediT authorship contribution statement

**Yuanfeng Li:** Conceptualization, Methodology, Software. **Yin-Zi Piao:** Visualization, Investigation. **Hua Chen:** Data curation, Resources. **Keqing Shi:** Visualization, Investigation. **Juqin Dai:** Investigation. **Siran Wang:** Software, Validation. **Tieli Zhou:** Resources, Investigation. **Anh-Tuan Le:** Software. **Yaran Wang:** Resources, Investigation. **Fan Wu:** Resources, Investigation. **Rujiang Ma:** Funding acquisition, Writing – review & editing. **Linqi Shi:** Supervision, Writing – review & editing. **Yong Liu:** Conceptualization, Funding acquisition, Supervision, Writing – review & editing.

## Declaration of competing interest

Y.L., Y.F.Li, and H.C. declare the following competing interests: a patent has been filed covering the materials and methods reported herein. All other authors declare no other competing interests.
