# Supplementary material for: Dynamic covalent nano-networks comprising antibiotics and polyphenols orchestrate bacterial drug resistance reversal and inflammation alleviation
Source: Bioact Mater. 2023 Apr 18;27:288–302. doi: 10.1016/j.bioactmat.2023.04.014 (PMC10126917; doi:10.1016/j.bioactmat.2023.04.014)
Supplement: Multimedia component 1 [file mmc1.zip › Supplementary files/Supporting Information-Bioactive materials-YFL.docx]

Supporting Information

Dynamic covalent nano-networks comprising antibiotics and polyphenols orchestrate bacterial drug resistance reversal and inflammation alleviation

Yuanfeng Li ^a,1^, Yin-Zi Piao ^b,1^, Hua Chen ^b,c,1^, Keqing Shi ^a^, Juqin Dai ^a^, Siran Wang ^b^, Tieli Zhou ^d,*^, Anh-Tuan Le ^e^, Yaran Wang ^b^, Fan Wu ^c^, Rujiang Ma ^c,*^, Linqi Shi ^c^, Yong Liu ^a,c,*^

^a^ Translational Medicine Laboratory, The First Affiliated Hospital of Wenzhou Medical University, Wenzhou, Zhejiang 325035, China. E-mail: y.liu@ucas.ac.cn (Y. Liu)

^b^ Wenzhou Institute, University of Chinese Academy of Sciences; Oujiang Laboratory (Zhejiang Lab for Regenerative Medicine, Vision and Brain Health); Wenzhou, Zhejiang 325001, China

^c^ Key Laboratory of Functional Polymer Materials of Ministry of Education, Institute of Polymer Chemistry, College of Chemistry, Nankai University, Tianjin, 300071, China. E-mail: marujiang@nankai.edu.cn (R. Ma)

^d^ Department of Clinical Laboratory, The First Affiliated Hospital of Wenzhou Medical University, Wenzhou, Zhejiang 325035, China. E-mail: [wyztli@163.com](mailto:wyztli@163.com)

^e^ Nano institute, Phenikaa University, Yen Nghia, Ha Dong, Ha Noi, Vietnam

^1^ Yuanfeng Li, Yin-Zi Piao, and Hua Chen contributed equally

Contents

[Experimental Section 3](#_Toc130995063)

[Materials, cells, and animals. 3](#_Toc130995064)

[Characterizations. 4](#_Toc130995065)

[Supplementary Figures 5](#_Toc130995066)

[Figure S1 5](#_Toc130995067)

[Figure S2 5](#_Toc130995068)

[Figure S3 6](#_Toc130995069)

[Figure S4 6](#_Toc130995070)

[Figure S5 7](#_Toc130995071)

[Figure S6 7](#_Toc130995072)

[Figure S7 8](#_Toc130995073)

[Figure S8 8](#_Toc130995074)

[Figure S9 9](#_Toc130995075)

[Figure S10 9](#_Toc130995076)

[Figure S11 9](#_Toc130995077)

[Figure S12 10](#_Toc130995078)

[Figure S13 10](#_Toc130995079)

[Figure S14 10](#_Toc130995080)

[Figure S15 11](#_Toc130995081)

[Figure S16 12](#_Toc130995082)

[Figure S17 12](#_Toc130995083)

[Figure S18 13](#_Toc130995084)

[Figure S19 14](#_Toc130995085)

[Figure S20 14](#_Toc130995086)

[Supplementary Tables 15](#_Toc130995087)

[Table S1. Summary of the stock solutions used in this study. 15](#_Toc130995088)

[Table S2. Sequences of genes used in this study. 15](#_Toc130995089)

[Table S3. Minimal inhibitory concentrations (MICs) and bactericidal concentrations (MBCs) of various treatments against *E. coli* WL5301 or *S. aureus* Xen36. 16](#_Toc130995090)

# Experimental Section

## Materials, cells, and animals.

*Chemicals and materials*: Polymyxins and aminoglycosides (USP grade) were purchased from Yuanyebio (Shanghai, China) or Macklin (Shanghai, China). Quercetin, epigallocatechin gallate (EGCG), ellagic acid (EA), tannic acid (TA), and phenylboronic acids were purchased from Macklin (Shanghai, China). Polyvinylpolypyrrolidone (PVP), *N*-methyl-2-pyrrolidone (NMP), dimethyl sulfoxide (DMSO), and other solvents were purchased from J&K Scientific (Beijing, China). 2',7'-dichlorodihydrofluorescein diacetate (H_2_DCFDA), 4,6-diamidino-2-phenylindole dihydrochloride (DAPI), SYTO™ 9, propidium iodide (PI), cell counting kit-8 (CCK-8) were purchased from Beyotime (Shanghai, China). RPMI 1640 medium, Dulbecco's Modified Eagle Medium (DMEM), penicillin-streptomycin solution, phosphate-buffered saline (PBS), and fetal bovine serum (FBS) for cell culture were purchased from Gibco Life Technologies, Inc. (Grand Island, NY, USA). Mouse IL-6, IL-10 ELISA kits, and macrophage colony-stimulating factor 1 (M-CSF) were purchased from iCell Bioscience Inc. (Shanghai, China). All chemicals were used without further purification.

*Bacterial strains*: Clinical isolate *E. coli* WL5301 (gifted from Yuhuan Hospital, Taizhou, China) and *S. aureus* Xen36 (PerkinElmer Inc., Waltham, MA, USA) were employed in this study. For experiments, one colony of *E. coli* WL5301 on lysogeny broth (LB) plates was inoculated into 10 mL of LB (OXOID, Basingstoke, UK) at 37 °C for 24 h in ambient air. This preculture was diluted at 1:20 in 200 mL of LB and grown statically for 16 h at 37 °C. *S. aureus* Xen36 was cultured on tryptic soy broth (TSB) agar plates with 200 μg/mL kanamycin. One colony was inoculated in 10 mL TSB (OXOID, Basingstoke, UK) and incubated for 24 h at 37 °C and used to inoculate (1:20) 200 mL main culture and grown for 16 h. Bacterial cultures were harvested by centrifugation at 5000*g* for 5 min, washed twice in PBS (5 mM K_2_HPO_4_, 5 mM KH_2_PO_4_, and 150 mM NaCl, pH 7.4), resuspended in 10 mL of PBS, and bacterial concentration was determined in a Bürker-Türk counting chamber.

*Cells:* Human embryonic kidney (HEK293) cells and murine macrophage cells (RAW 264.7) were obtained from the American Type Culture Collection. HEK293 were cultured in a DMEM containing 10% FBS and 1% penicillin−streptomycin solution. RAW 264.7 cells were cultured in an RPMI 1640 medium containing 10% FBS. Bone-marrow-derived macrophages (BMDMs) were isolated from female ICR mice. Briefly, one ICR mouse was sacrificed by cervical dislocation and disinfected with 75% alcohol. The hind legs were cut off, and the flesh and muscles adhering to the bones were removed. The tibias were cut off from the femur at the joint with sterile scissors, and the cells in the bone were collected by flushing the bones with PBS using a 1 mL syringe. The pellet was collected through a 70 µm strainer and centrifuged at 800*g* for 5 min at 4 °C. The bottom pellet was collected and lysed in 5 mL red blood cell lysis buffer for 5 min. The bottom pellet was collected by centrifuge and cultured in RPMI 1640 medium containing 10% FBS and 20 ng/mL M-CSF. Half of the medium was replaced by freshly RPMI 1640 medium containing M-CSF after two days. The cells were used for experiments after seven days.

*Animals*: Female ICR mice (6-8 weeks, 18-20 g) were purchased from Zhejiang Vital River Laboratory Animal Technology Co., Ltd and were housed in an SPF room. The animal experimental protocols were reviewed and approved by the Institutional Animal Care and Use Committee, Wenzhou Institute, University of Chinese Academy of Sciences (No. WIUCAS21071223).

## Characterizations.

The size and zeta potential of these freshly prepared aDCNs were measured on a Zetasizer Nano ZEN3600 (Malvern, UK) with aDCNs diluted in phosphate buffer (10 mM, pH 7.4) to a concentration of 100 µg/mL. The morphologies of aDCNs were observed on a Talos F200S (Thermo Scientific, US) transmission electron microscope. The UV-Vis spectra of aDCNs and other solutions/formulations were recorded on a UV-1900i spectrometer (Shimadzu, Japan). For nuclear magnetic resonance (NMR) and Fourier transform infrared spectroscopy (FT-IR) measurements, aDCNs were prepared without the addition of PVP according to the procedure described above. Then, solid-state NMR spectroscopy was measured on a Bruker AVANCE III 400 MHz spectrometer using 1.33 mm probe to get 40kHz MAS spectra (Bruker, Germany) at 25 °C, and FT-IR spectra were recorded on a Tensor II spectrometer (Bruker, Germany).

# Supplementary Figures

## Figure S1

Size distribution and PDI of the as-prepared **A1B1C1** nano-networks.


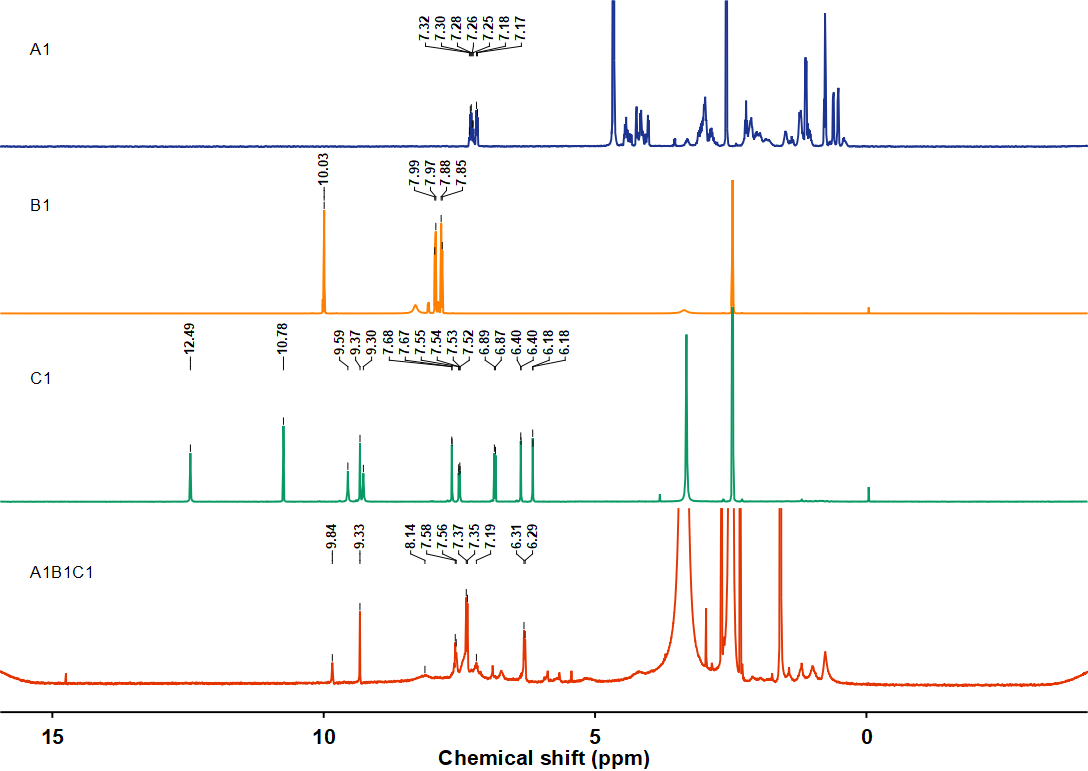


## Figure S2

^1^H NMR spectra of **A1** in D_2_O, **B1**, **C1**, and **A1B1C1** in solution. **A1** was dissolved in D_2_O. **B1**, **C1** and **A1B1C1** was measured in *d*_6_-DMSO.


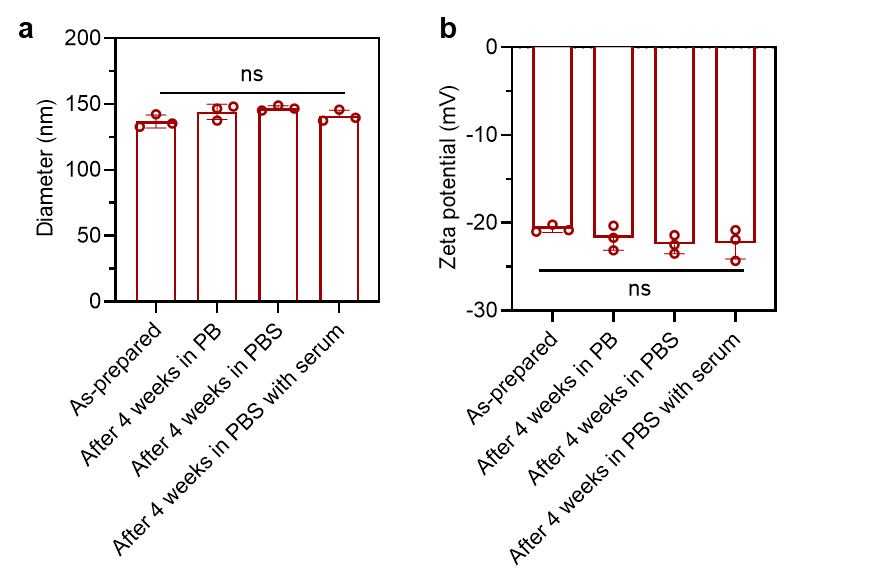


## Figure S3

Size (**a**) and zeta potential (**b**) of the as-prepared nano-networks and the nano-networks after 4 weeks in PB, PBS with or without 10% FBS.

## Figure S4

UV-Vis absorption curve of the as-prepared **A1C1** in solution and **A1C1** solution after 24 h storage at room temperature.

## Figure S5

Size distribution of the **A1B1C1** nano-networks after three-week storage at room temperature.

## Figure S6

Thermal analysis data of various formulations.


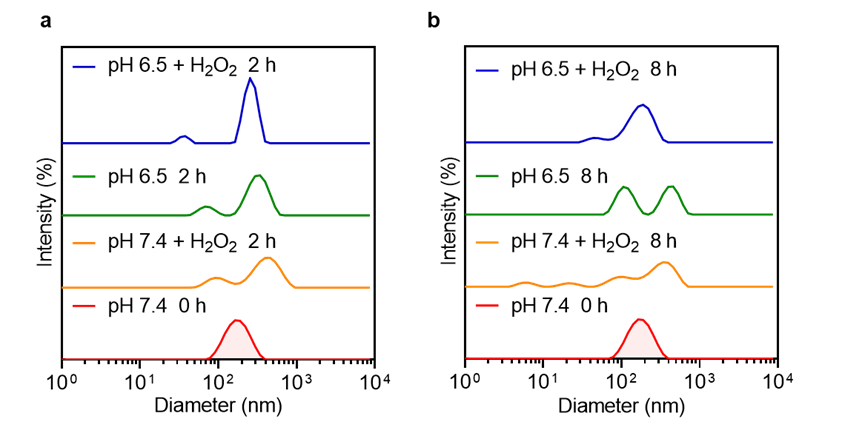


## Figure S7

(**a**) Size distribution of the **A1B1C1** nano-networks after various treatments for 2 h. (**b**) Size distribution of the **A1B1C1** nano-networks after various treatments for 8 h.


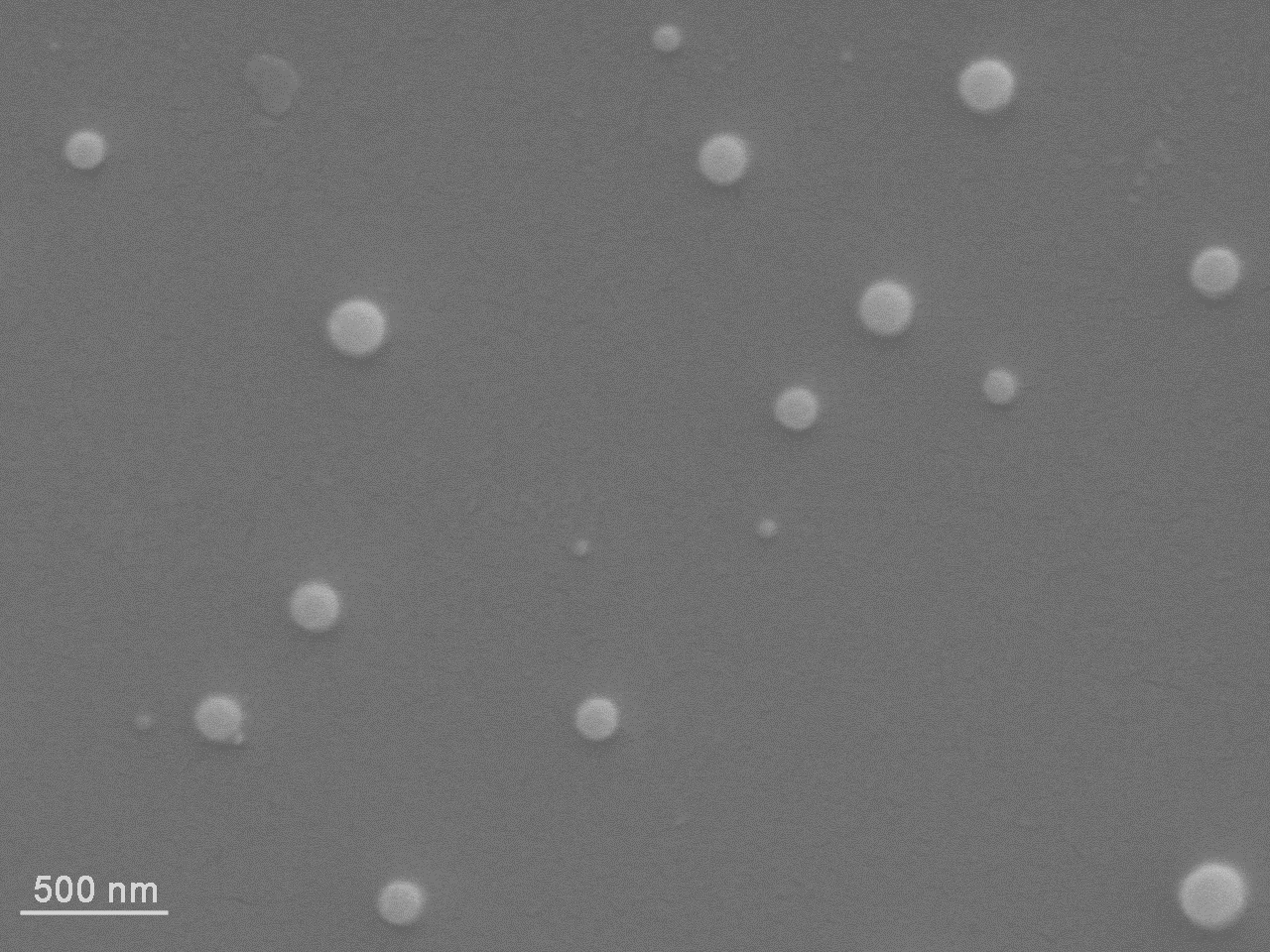


## Figure S8

SEM image of the as-prepared **A1B1C1** nano-networks.


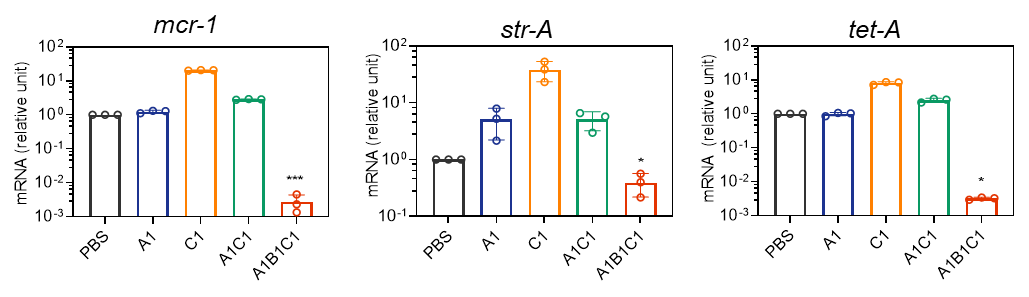


## Figure S9

Relative gene expression in *E. coli* WL5301 after various treatments. Gene expression in bacteria treated with PBS was used as control. * *p* < 0.05, *** *p* < 0.001, one-way ANOVA.


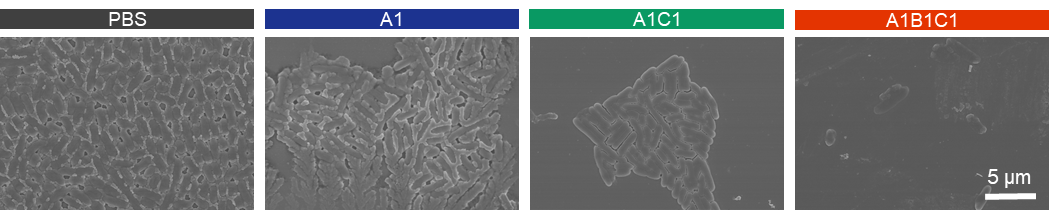


## Figure S10

Representative SEM images of *E. coli* WL5103 after various treatments.

## Figure S11

Relative expression of *fimH* gene in *E. coli* WL5301 after various treatments. Gene expression in bacteria treated with PBS was used as control. * *p* < 0.05, one-way ANOVA.

## Figure S12

Fluorescence intensity of the PI-stained eDNA from culture medium of *E. coli* WL5301 in the presence of various treatments. **** *p* < 0.0001, one-way ANOVA.


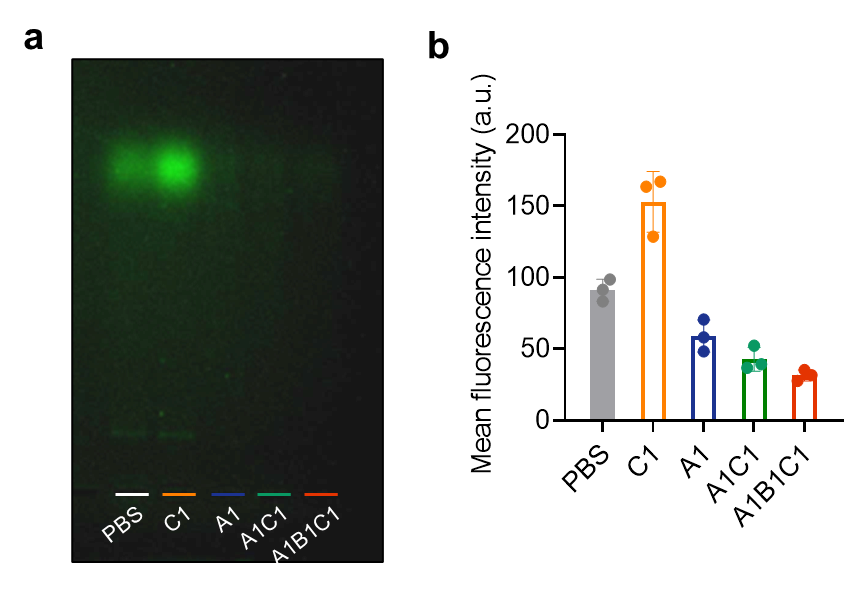


## Figure S13

(**a**) Agarose gel electrophoresis image of eDNA from culture medium of *E. coli* WL5301 in the presence of various treatments. (**b**) Mean fluorescence intensity quantified from panel **a**.


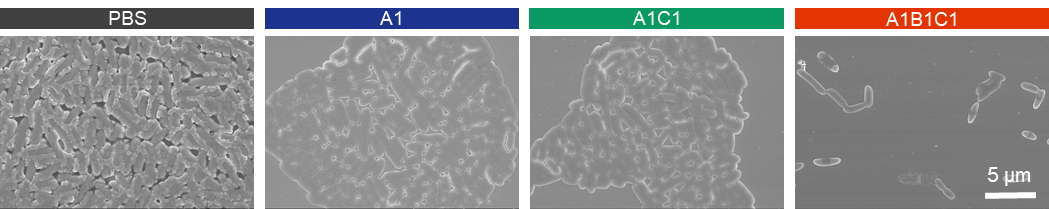


## Figure S14

Representative SEM images of *E. coli* WL5103 biofilms after various treatments.

**
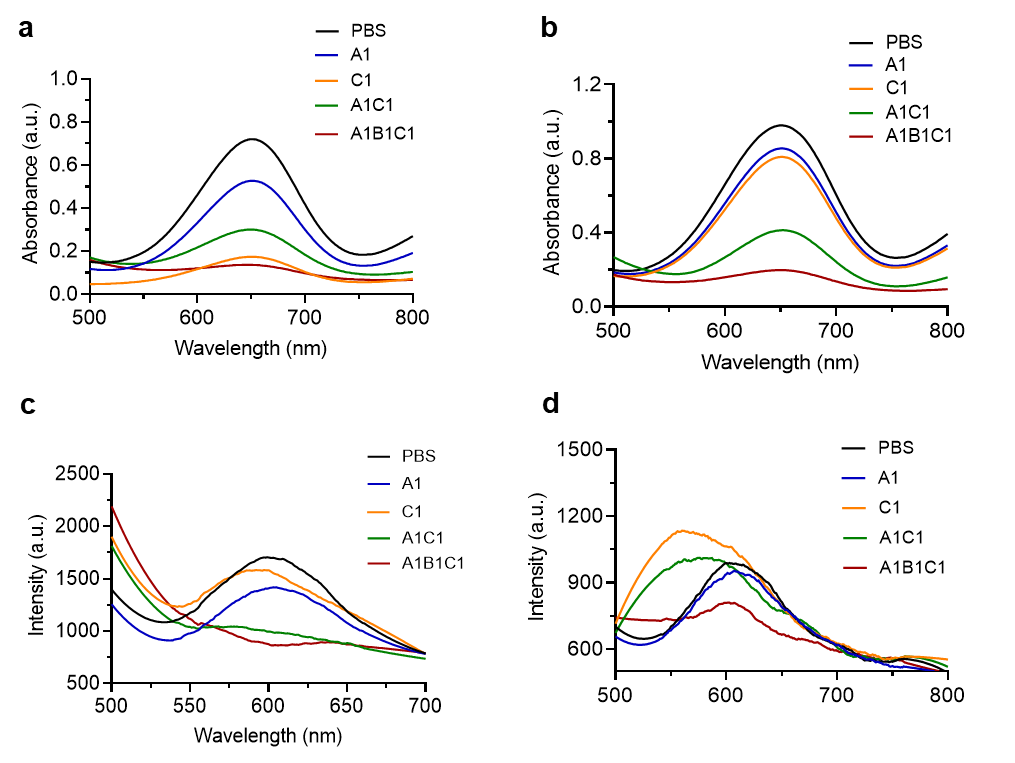
**

## Figure S15

(**a**) UV-vis absorption of TMB after the treatments with various as-prepared formulations in the presence of Cu^2+^/H_2_O_2_. (**b**) UV-vis absorption of TMB after the treatments with various formulations stored for 24 h in the presence of Cu^2+^/H_2_O_2_. (**c**) Fluorescent intensity of ethidium (the oxidation product of hydroethidine by superoxide radical) after the treatments with various as-prepared formulations. (**d**) Fluorescent intensity of ethidium (the oxidation product of hydroethidine by superoxide radical) after the treatments with various formulations stored for 24 h.


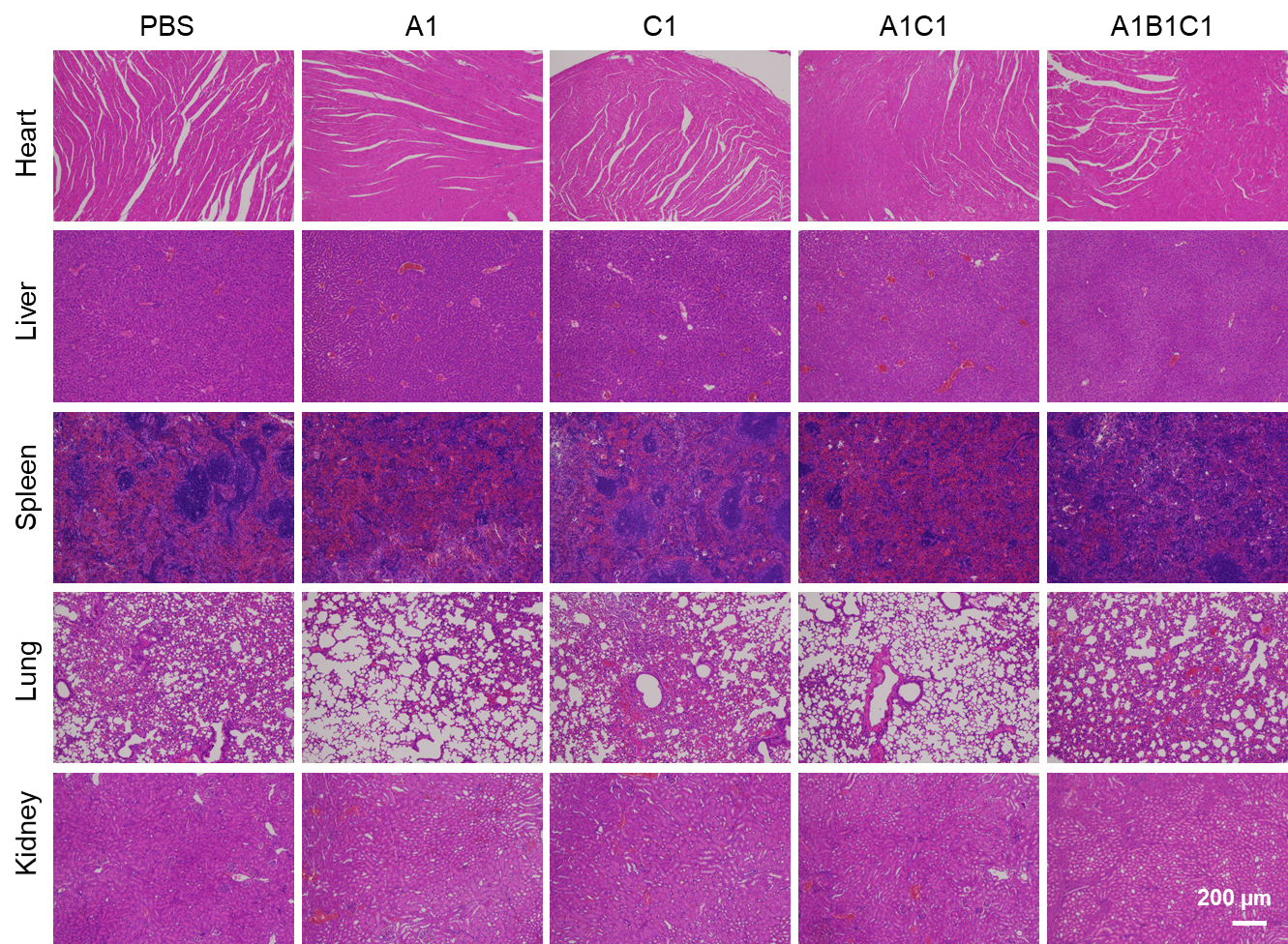


## Figure S16

H&E staining of major organ sections harvested from mice after various treatments on day 5 in the peritoneal infection model. Bar represents 200 μm.


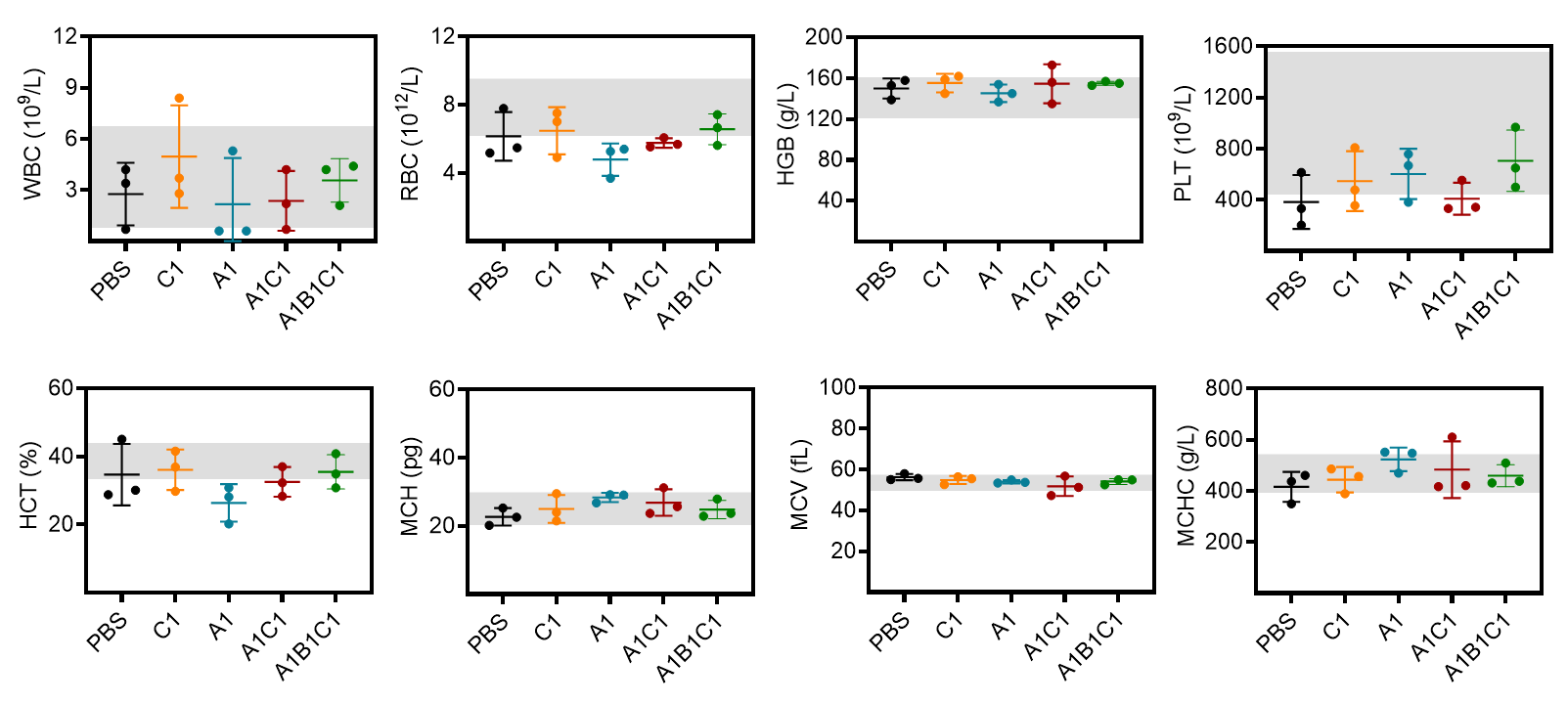


## Figure S17

Major blood parameters mice after various treatments. Blood samples were taken on day 2 after initiating treatment. White blood cell is WBC, red blood cell RBC, hemoglobin Hgb, platelet Plt, hematocrit Hct, mean corpuscular hemoglobin MCH, mean corpuscular volume MCV, and mean corpuscular hemoglobin concentration is MCHC. Error bars denote SD over three mice in each group.


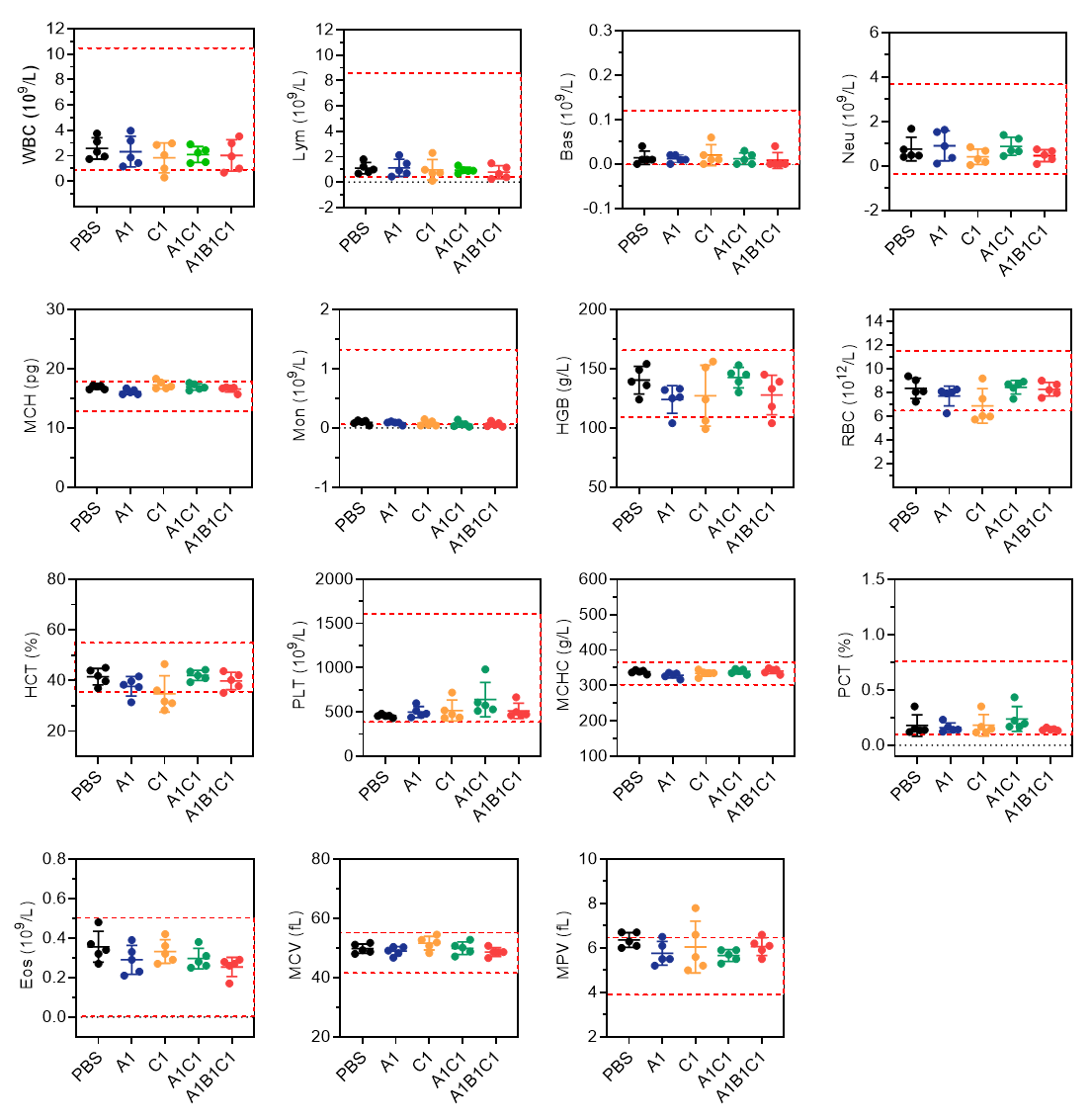


## Figure S18

Major blood parameters mice after various treatments in the biofilm-associated infection model. Blood samples were taken on day 2 after initiating treatment. White blood cell is WBC, Lymphocytes Lym, basophil bas, neutrophils Nue, mean corpuscular hemoglobin MCH, Monocytes Mon, hemoglobin HGB, red blood cell RBC, hematocrit HCT, platelet PLT, mean corpuscular hemoglobin concentration MCHC, platelet count PCT, Eosinophils Eos, mean corpuscular volume MCV, mean platelet volume is MPV. Error bars denote SD over three mice in each group.

## Figure S19

Change of body weight over the 7-d observation post-treatment in the biofilm-associated infection model (n = 6).


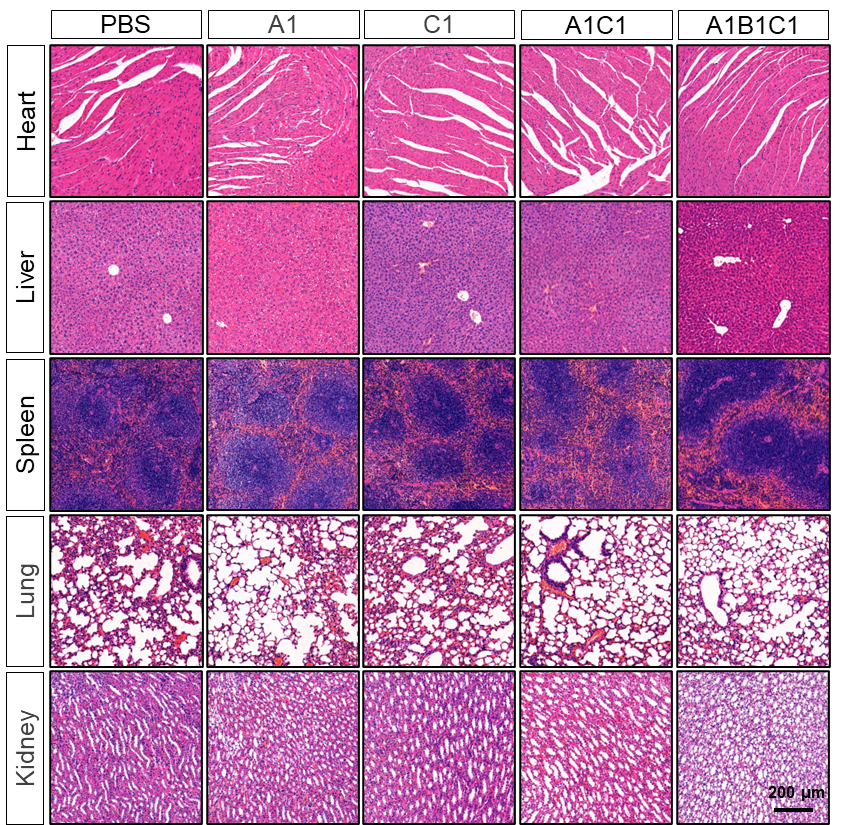


## Figure S20

H&E staining of major organ sections harvested from mice after various treatments on day 7 in the implant infection model. Bar represents 200 μm.

# Supplementary Tables

## Table S1. Summary of the stock solutions used in this study.

| Chemicals | Solvent | Concentration (mg/mL) |
| --- | --- | --- |
| Polymyxin B | Ultrapure water | 10 |
| Aminoglycosides | Ultrapure water | 10 |
| Quercetin | DMSO | 10 |
| Epigallocatechin gallate | Ultrapure water | 10 |
| Ellagic acid | NMP | 10 |
| Tannic acid | Ultrapure water | 10 |
| Aldehyde phenylboronic acids | DMSO | 10 |

## Table S2. Sequences of genes used in this study.

| Genes | Description | Sequence |
| --- | --- | --- |
| *mcr-1* | Forward | 5’-CGCTGTTATCATCGTATCG-3’ |
|  | Reverse | 5’-GTATCATAGACCGTGCCATA-3’ |
| *strA* | Forward | 5’-CCTGGTGATAACGGCAATTC-3’ |
|  | Reverse | 5’-CCAATCGCAGATAGAAGGC-3’ |
| *tetA* | Forward | 5’-CGGTCTTCTTCATCATGCAAC-3’ |
|  | Reverse | 5’-GTCCCAGTGAAAGCGATCC-3’ |
| *fimH* | Forward | 5’-TCGAGAACGGATAAGCCGTGG-3’ |
|  | Reverse | 5’-GCAGTCACCTGCCCTCCGGTA-3’ |
| 16s rDNA | Forward | 5’- ACTCCTACGGGAGGCAGCAGT-3’ |
|  | Reverse | 5’-TATTACCGCGGCTGCTGGC-3’ |

## Table S3. Minimal inhibitory concentrations (MICs) and bactericidal concentrations (MBCs) of various treatments against *E. coli* WL5301 or *S. aureus* Xen36.

| **Strains** | **Entry** | **MIC (µg mL^-1^)** | **MBC (µg mL^-1^)** |
| --- | --- | --- | --- |
|  | PVP | > 500 | > 500 |
|  | B1-B6 | > 500 | > 500 |
|  | C1-C4 | > 500 | > 500 |
| *E. coli* WL5301 | A1 | 32 | 32 |
|  | A1B1C1 | 16 | 32 |
|  | A1B1C2 | 32 | 64 |
|  | A1B1C3 | 32 | 64 |
|  | A1B1C4 | 16 | 32 |
|  | A1B2C1 | 16 | 16 |
|  | A1B3C1 | 16 | 32 |
|  | A1B4C1 | 16 | 16 |
|  | A1B5C1 | 16 | 32 |
|  | A1B6C1 | 16 | 32 |
| *S. aureus* Xen36 | A2 | 2 | 4 |
|  | A2B1C2 | 2 | 4 |
|  | A2B2C2 | 2 | 4 |
|  | A2B3C2 | 2 | 4 |
|  | A2B4C2 | 2 | 4 |
|  | A2B5C2 | 2 | 2 |
|  | A2B6C2 | 2 | 2 |
|  | A2B6C1 | 2 | 4 |
|  | A2B6C3 | 2 | 4 |
|  | A2B6C4 | 2 | 2 |
|  | A3 | 32 | >64 |
|  | A3B6C1 | 32 | 64 |
|  | A3B6C2 | 16 | 32 |
|  | A3B6C3 | 32 | 64 |
|  | A3B6C4 | 32 | 32 |
|  | A4 | 4 | 8 |
|  | A4B6C1 | 2 | 4 |
|  | A4B6C2 | 2 | 4 |
|  | A4B6C3 | 4 | 16 |
|  | A4B6C4 | 16 | 16 |
|  | A5 | 16 | 32 |
|  | A5B6C1 | 16 | 32 |
|  | A5B6C2 | 32 | 32 |
|  | A5B6C3 | 16 | 16 |
|  | A5B6C4 | 4 | 8 |
